# Phytoextraction of rare earth elements in herbaceous plant species growing close to roads

**DOI:** 10.1007/s11356-017-8944-2

**Published:** 2017-04-14

**Authors:** Patrycja Mikołajczak, Klaudia Borowiak, Przemysław Niedzielski

**Affiliations:** 1 Department of Ecology and Environmental Protection, Poznan University of Life Sciences, Piątkowska 94C, 60-649 Poznań, Poland; 2 Faculty of Chemistry, Adam Mickiewicz University in Poznań, Umultowska 89B, 61-614 Poznań, Poland

**Keywords:** Rare earth elements, Phytoextraction, Herbaceous plants, Traffic pollution

## Abstract

The aim of study was to determine the phytoextraction of rare earth elements (REEs) to roots, stems and leaves of five herbaceous plant species (*Achillea millefolium* L., *Artemisia vulgaris* L., *Papaver rhoeas* L., *Taraxacum officinale* and *Tripleurospermum inodorum*), growing in four areas located in close proximity to a road with varied traffic intensity. Additionally, the relationship between road traffic intensity, REE concentration in soil and the content of these elements in plant organs was estimated. *A. vulgaris and P. rhoeas* were able to effectively transport REEs *in their leaves, independently of area collection. The highest content of REEs* was observed *in P. rhoeas* leaves and *T. inodorum* roots. Generally, HREEs were accumulated in *P. rhoeas* roots and leaves and also in the *stems of T. inodorum* and *T. officinale, whereas LREEs were accumulated in T. inodorum* roots and *T. officinale stems. It is worth underlining that there was a clear relationship between road* traffic intensity and REE, HREE and LREE concentration in soil. No positive correlation was found between the concentration of these elements in soil and their content in plants, with the exception of *T. officinale.* An effective transport of REEs from the root system to leaves was observed, what points to the possible ability of some of the tested plant species to remove REEs from soils near roads.

**Electronic supplementary material:**

The online version of this article (doi:10.1007/s11356-017-8944-2) contains supplementary material, which is available to authorized users.

## Introduction

There are only a relatively low number of investigations concerning rare earth elements (REEs) in comparison to other groups of elements characterized in various environmental components (Tyler 2004; Bentlin and Pozebon 2010; Zhang et al. 2013; Mleczek et al. 2016a,b;). This is mainly connected with analytical problems, both the lack of proper equipment and lack of reference materials (Cao et al. 2000). REEs, contrary to commonly held opinion, are not a rarely occurring group of elements in the environment. This has been proved e.g. by investigations on neodymium (Nd) or scandium (Sc). The content of REEs in the environment is increasing due to the development of modern technologies and a consequent elevated need for selected elements concerning the type of final product (EPA 600/R-12/572 2012). REEs are important in many branches of the economy, such as agriculture, the petroleum industry, applications in television and information technology. The use of rare elements in many products improves the features of other elements by increasing their resistance, conductivity, strength and decreasing the weight and size of the final product (Klinger 2015).

According to Klinger (2015), there are 17 rare earth elements which represent approx. 17% of all elements occurring in the natural environment. On the other hand, Gambogi (2011) divided REEs into two groups: heavy rare earth elements (HREEs) and light rare earth elements (LREEs). The following are considered to be LREEs: scandium (^21^Sc), yttrium (^39^Y), terbium (^65^Tb), dysprosium (^66^Dy), holmium (^67^Ho), erbium (^68^Er), thulium (^69^Tm), ytterbium (^70^Yb) and lutetium (^71^Lu), while HREEs are: lanthanum (^57^La), cerium (^58^Ce), praseodymium (^59^Pr), neodymium (^60^Nd), promethium (^61^Pm), samarium (^62^Sm), europium (^63^Eu) and gadolinium (^64^Gd). Other researchers have described REEs as a group of 14 elements belonging to lanthanides beginning from ^57^La to ^71^Lu (Laveuf and Cornu 2009).

The concentration of REEs has been analysed in soil (Laveuf and Cornu 2009), water cultures (Ding et al. 2005), and in plants (Nakamaru et al. 2006; Bluemel et al. 2013; Zhang et al. 2015). In the case of plants, REEs were characterized with respect to the possibility of selecting ligands for complexation of these elements, and modulation of their phytoextraction efficiency, both from soil and water cultures (Byrne et al. 1996; Ding et al. 2005). REEs have been analysed in numerous types of plants: lichens, mosses or vascular plants (Chiarenzelli et al. 2001), such as maize plants (Saatz et al. 2015) or the functional group of grasses (Wiche and Heilmeier 2016). The general characteristics of REE content in numerous components of the environment is presented in Supplemementary data (Table S1).

The use of automotive catalytic converters for almost 50 years has increased the amount of platinum group elements (PGE). On the other hand, some authors have focused on numerous other elements, such as REEs (Djingova et al. 2003). The increase in their concentration in soil can be associated with the rapid development of new technologies (Carrero et al. 2013). Owing to the transport of these elements and their implementation in the production of selected parts of cars, their content in soil and accumulation in plants that grow close to roads has increased (Figueiredo et al. 2009).

For this reason, the aim of present study was to evaluate: *i)* the efficiency of REEs phytoextraction to roots, stems and leaves of *Achillea millefolium* L., *Artemisia vulgaris* L., *Papaver rhoeas* L., T*araxacum officinale* G.H. Wigg. and *Tripleurospermum inodorum* L. samples collected from four different locations, *ii)* the similarities and differences between plant species and *iii)* the relationship between road traffic intensity, REEs concentration in soil and the content of these elements in organs of the tested plants collected from all experimental areas.

## Materials and methods

### Experiment design

**Fig. 1:**
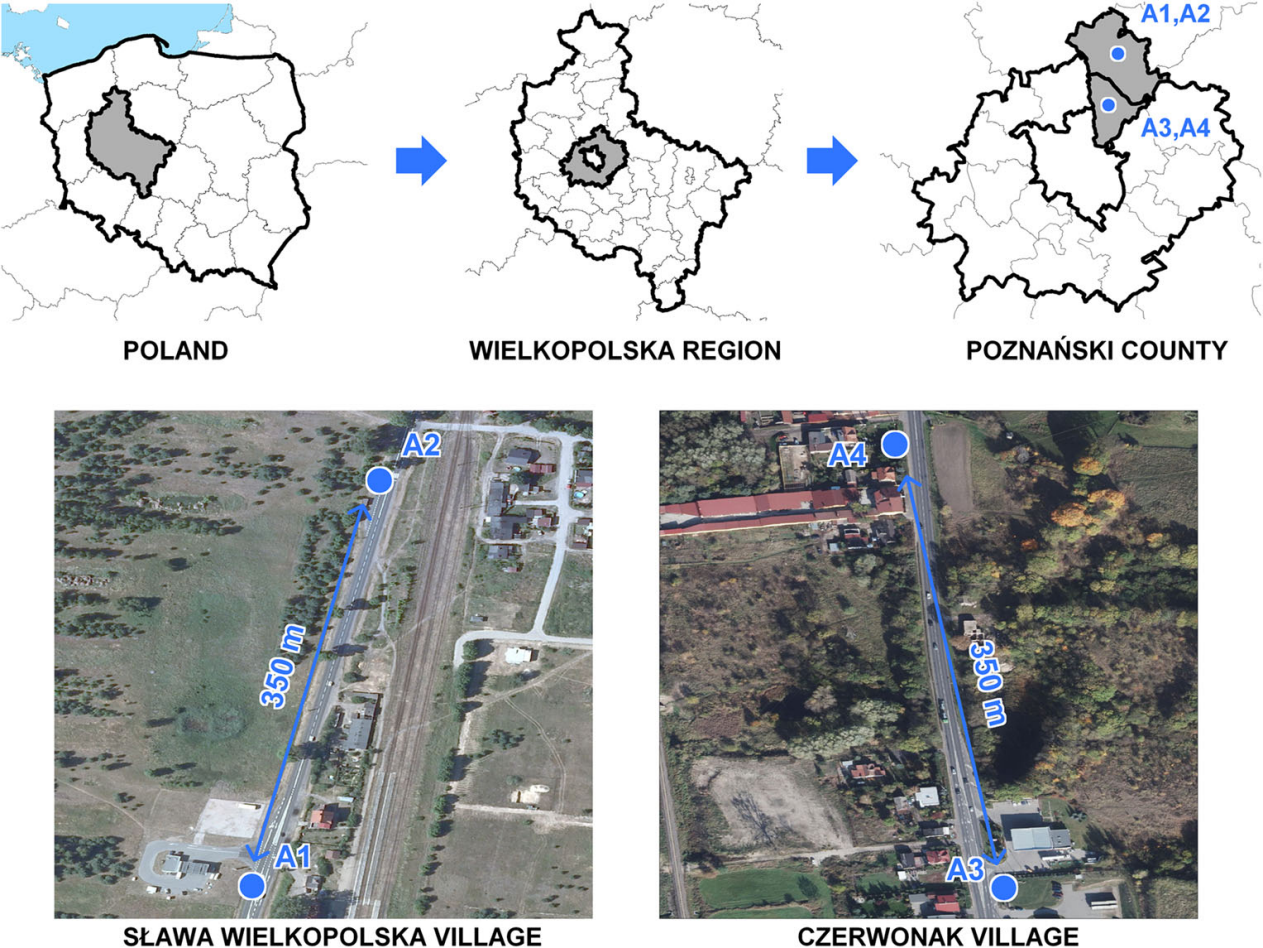
Map of the sampled areas

Plant samples were collected from near the road and in the vicinity of petrol stations located in the villages of Sława Wielkopolska (Areas A1 and A2) and Czerwonak (Areas A3 and A4). In this study, five herbaceous plant species, growing at four areas located along roads 196 and 31, 5 km north of the city of Poznań (Western Poland), were collected. Particular experimental areas were diverse as regards traffic intensity (Table 1) and their location at petrol stations, or within approximately 400 m of them (Fig. 1).

**Table 1:** Characteristics of motor vehicles intensity at experimental areas

Mean amount of motor vehicles	2015				2016			
	Area 1 PS*	Area 2	Area 3 PS*	Area 4	Area 1 PS*	Area 2	Area 3 PS*	Area 4
	9655	9472	17,438	17,097	9716	9503	17,699	17,204
Motocycles	106	99	206	204	75	72	227	197
Passengercars	7948	7860	14,344	14,135	8132	7943	14,792	14,212
Delivery vans	790	735	1423	1343	804	766	1469	1423
Lorries	695	677	1256	1211	602	626	1244	1243
Buses	87	72	157	157	82	66	127	90
Tractors	29	29	52	47	21	30	67	39

*PS – nearby petrol station

The data presented in Table 1 were obtained between 8th and 11th August 2015 and 2016, respectively, when the number of passing motor vehicles was counted at each experimental area between 8 a.m. and 8 p.m.

### Experimental materials

Soil samples were collected from the topsoil (to maximal depth of 15 cm) to the amount of 0.5 kg, which was an average from 15 samples of soil collected using a sand auger (AMS, Inc.) with a diameter of 3.8 cm (1.5″). The characteristics of the experimental areas are presented in Table 2.

**Table 2:** Characteristics of experimental areas of plant samples collection with data of elements concentration [mg kg^−1^ DW] in soil

Data	Unit	Sława Wielkopolska village		Czerwonak village	
		petrol station	road	petrol station	road
		Area 1 (A1)	Area 2 (A2)	Area 3 (A3)	Area 4 (A4)
GPS* position		52°38′3.86″N 17°8′35.25″E	52°38′15.50″N 17° 8′39.05″E	52°27′25.52″N 16°59′3.27″E	52°27′37.94″N 16°59′1.48″E
Elevation*	m	78	79	62	64
Element		Chemical characteristics of soil			
P	mg kg^−1^	505^a^ ± 61	416^a^ ± 11	468^a^ ± 39	440^a^ ± 20
S	%	0.021^a^ ± 0.002	0.023^a^ ± 0.002	0.019^a^ ± 0.002	0.020^a^ ± 0.001
Ca	mg kg^−1^	1240^b^ ± 30	1210^b^ ± 90	9540^a^ ± 690	9520^a^ ± 250
K	mg kg^−1^	400^b^ ± 23	456^b^ ± 34	983^a^ ± 30	866^a^ ± 63
Mg	mg kg^−1^	554^b^ ± 18	606^b^ ± 113	1210^a^ ± 90	1160^a^ ± 60
Na	mg kg^−1^	94^b^ ± 10	118^ab^ ± 17	142^ab^ ± 18	150^a^ ± 21
		LREEs			
Ce	mg kg^−1^	19.3^b^ ± 1.6	19.5^b^ ± 1.8	25.9^a^ ± 0.8	24.2^a^ ± 1.3
Eu		0.19^ab^ ± 0.02	0.15^b^ ± 0.01	0.23^a^ ± 0.02	0.22^a^ ± 0.03
Gd		0.24^b^ ± 0.01	0.21^b^ ± 0.02	1.91^a^ ± 0.06	1.86^a^ ± 0.15
La		1.02^b^ ± 0.03	0.95^b^ ± 0.03	6.75^a^ ± 0.23	6.27^a^ ± 0.46
Nd		7.31^b^ ± 0.07	7.75^b^ ± 0.68	14.1^a^ ± 0.8	13.4^a^ ± 0.4
Pr		1.01^b^ ± 0.05	1.06^b^ ± 0.13	1.80^a^ ± 0.16	1.55^a^ ± 0.11
Sm		0.04^a^ ± 0.02	0.04^a^ ± 0.01	0.03^a^ ± 0.02	0.05^a^ ± 0.01
		HREEs			
Dy	mg kg^−1^	0.90^a^ ± 0.02	0.83^a^ ± 0.06	0.64^b^ ± 0.04	0.65^b^ ± 0.02
Er		28.5^b^ ± 2.4	30.8^b^ ± 1.3	48.1^a^ ± 3.0	45.9^a^ ± 1.1
Ho		bDL**	bDL	0.04^a^ ± 0.02	0.04^a^ ± 0.01
Lu		0.11^c^ ± 0.02	0.12^c^ ± 0.01	0.19^a^ ± 0.03	0.18^b^ ± 0.01
Sc		0.41^b^ ± 0.02	0.40^b^ ± 0.02	0.94^a^ ± 0.04	0.88^a^ ± 0.03
Tb		0.11^b^ ± 0.02	0.13^b^ ± 0.02	0.26^a^ ± 0.03	0.27^a^ ± 0.02
Tm		0.41^c^ ± 0.03	0.45^c^ ± 0.02	1.20^a^ ± 0.03	1.05^b^ ± 0.03
Yb		0.26^b^ ± 0.02	0.28^b^ ± 0.02	0.38^a^ ± 0.02	0.36^a^ ± 0.03
Y		1.79^b^ ± 0.15	1.93^b^ ± 0.05	2.68^a^ ± 0.04	2.53^a^ ± 0.08
pH		6.29^ab^ ± 0.02	6.35^a^ ± 0.01	6.21^b^ ± 0.03	6.25^b^ ± 0.04
EC	mS cm^−1^	498^bc^ ± 24	464^c^ ± 14	623^a^ ± 11	555^b^ ± 23
Redox	eV	218^a^ ± 14	216^a^ ± 13	258^a^ ± 18	238^a^ ± 22

*determined using the Global Positioning System (GPS) by GPS Navigator Oregon 550 t (Garmin, USA)

**bDL–below Detection Limit

Experimental material consisted of 5 popular herbaceous plant (weed) species: *Achillea millefolium* L., *Artemisia vulgaris* L., *Papaver rhoeas* L., *Tripleurospermum inodorum* (L.) Sch. Bip. and *Taraxacum officinale* F. H. Wigg., selected on the grounds of their common presence in the environment, growth in close proximity to the road at the experimental areas, resistance to unfavourable weather conditions and rapid growth. With the exception of *P. rhoeas* L., which belongs to the *Papaveraceae* family, the other four plant species belong to the *Asteraceae* family. Selection of the just plant species was an effect of their presence of the each year and possibility to collect the plants with the similar biomass. The mean biomass [g] of collected herbaceous plant species were as follow: 2.535 ± 0.475 (*A. millefolium*), 26.036 ± 4.063 (*A. vulgaris*), 17.065 ± 2.479 (*T. inodorum*), 11.005 ± 2.560 (*P. rhoeas*) and 4.385 ± 1.189 (*T. officinale*) with the percentage share of biomass of roots in whole plants biomass: 23.3; 14.2; 27.2; 39.3 and 33.2%, respectively.

### Sample collection and preparation

All weed species were collected from four experimental areas (*n* = 4) as 4 individual plants from each area on 12th August 2015 in drought conditions after a period of 13 days without rainfall, and 13th August 2016 after some rainy days (Supplementary data, Table S2). Both soil and plants were collected within 1–2 m distance of the road. Each plant was divided into three organs (roots, stems and leaves), dried using an electric oven TC 100 (SalvisLAB, Switzerland) at 105 ± 3 °C for 96 h and ground for 3 min in a Cutting Mill SM 200 (Retsch, Germany). Before drying roots and leaves were washed in deionized water to remove remaining soil particles from the root systems and any surface dust absorbed by leaves that could contain REEs ions. Three samples prepared for each plant organ were digested using a microwave mineralization system CEM Mars 5 Xpress (*CEM*, USA). 0.3000 ± 0.0001 g of sample was placed in 55 mL vessels with 7 mL of concentrated (65%) HNO_3_ and 1 mL of H_2_O_2_ (both of analytical purity, Merck, Germany). The digestion programme consisted of the following stages: first stage −10 min at power 600 W, temperature 80 °C; second stage – 10 min at power 1200 W, temperature 120 °C; third stage – 12 min at power 1600 W, temperature 200 °C. After digestion the solutions were filtrated through Qualitative Filter Papers (Grade 595: 4–7 μm Whatman, GB) and filled (to protect the analytical instrument) with deionized water Milli-Q Advantage A10 Water Purification Systems (Merck Millipore, Germany) to a final volume of 50 mL. Soil samples were digested in the same way as plant material but after mercerization for 24 h.

### Analytical methods

REEs determination was carried out using inductively coupled plasma optical emission spectrometry (ICP-OES) with an Agilent 5100 (Agilent, USA) spectrometer. The dual (synchronous) axial and radial plasma view was used. The instrumental parameters common for determination of all elements were: RF power 1.2 kW, plasma gas (argon) flow 12 L min^−1^, nebuliser gas (argon) flow 0.7 L min^−1^, radial view height 8 mm. For REE determination the following wavelengths were used: Ce 446.021 nm; Dy 400.045 nm; Er 349.910 nm; Eu 420.504 nm; Gd 342.246 nm; Ho 348.484 nm; La 333.749 nm; Lu 307.760 nm; Nd 406.108 nm; Pr 417.939 nm; Sc 361.383 nm; Sm 442.434 nm; Tb 350.914 nm; Tm 336.261 nm; Y 361.104 nm; Yb 328.937 nm .

Analysis of pH, redox potential, electrolytic conduction, and dry matter to characterize soils from particular areas were performed according to Polish and European norms (PN-ISO 10390 1997, ISO 11271 2002, PN-ISO 1265+AC1 1997 and PN-ISO 11465 1999, respectively). For pH, redox potential and electrolytic conduction the multiparametric system 350 (WTW, Germany) has been used.

### Control of quality of results

In traceability control the certified standard material CRM NCSDC (73349) – bush branches and leaves was applied (NACIS, China). The recovery values were: Ce 119%; Dy 77%; Eu 77%; Gd 105%; Ho 82%; La 87%; Lu 91%; Nd 110%; Pr 83%; Sm 118%; Yb 79% respectively. Because the Er and Sc concentration was not certified, an analysis of the certified standard material CRM 667 (sediment) was provided additionally (IRMM, Belgium). The obtained recoveries were: Er 105% and Sc 107%. The recovery values in the range of 75–125% were recognized as satisfied.

### Statistical analysis

The results were analysed with a factorial ANOVA with “plant species” and “plant organ” as fixed factors. Tukey’s test was employed to analyse differences between measured parameters. A graphical presentation of Tukey’s test results is provided in the present study. To determine the structure and relations between variables, principal component analysis (PCA) was used. In this analysis the orthogonal transformation of observed variables to a new set of non-correlated variables (components) is performed.

For the analyses of similarities and differences of REEs accumulation in certain plants within the experimental areas in regard to transport intensity and petrol station location cluster analysis was performed. Euclidean distance measures and Ward hierarchical clustering were used to determine the dendrogram with two or three groups. The certain Euclidean distance measure can designate a similarity structure in interactions between analysed parameters. The cluster analysis was carried out for LREE and HREE separately. The above data analyses were done with the statistical software STATISTICA 12.1 (StatSoft, USA).

To characterize the phytoextraction efficiency of LHREEs, HREEs and REEs, the bioconcentration factor (BCF) values were calculated according to eq. 1, as the ratio of the concentration of these elements in plant harvested organs to their concentration in soil (Ali et al. 2013).

1 $$ \mathrm{BCF}=\frac{\mathrm{LREEs}/\mathrm{HREEs}/\mathrm{REEs}\ \mathrm{concentration}\ \mathrm{in}\ \mathrm{the}\ \mathrm{root}\ \left(\mathrm{mg}\ {\mathrm{kg}}^{-1}\ \mathrm{DW}\right)}{\mathrm{LREEs}/\mathrm{HREEs}/\mathrm{REEs}\ \mathrm{concentration}\ \mathrm{in}\ \mathrm{soil}\ \left(\mathrm{mg}\ {\mathrm{kg}}^{-1}\ \mathrm{DW}\right)} $$

Moreover, to show the efficiency of REEs transport from roots to stems, the Translocation Factor (TF) values were calculated according to Yu and Zhou (2009) and the following formula (eq. 2).

2 $$ \mathrm{TF}=\frac{\mathrm{LREEs}/\mathrm{HREEs}/\mathrm{REEs}\ \mathrm{content}\ \mathrm{in}\ \mathrm{stems}\ \left(\mathrm{mg}\ {\mathrm{kg}}^{-1}\ \mathrm{DW}\right)}{\mathrm{LREEs}/\mathrm{HREEs}/\mathrm{REEs}\ \mathrm{content}\ \mathrm{in}\ \mathrm{roots}\ \left(\mathrm{mg}\ {\mathrm{kg}}^{-1}\ \mathrm{DW}\right)} $$

## Results

The results obtained in this study were prepared as preliminary mean values calculated for two years of studies performed in the environment. With respect to the same relations between the tested phytoextraction ability of plant species for REEs in 2015 and 2016 and the difference in the level of these elements in plant organs analysed in particular years, the results presented below are characteristic of materials analysed in 2015 only, with the appropriate comments in the final part of this section.

### Total content of rare earth elements in plant organs

The total amount of REEs is presented in Table 3 to illustrate differences in the effectiveness of their phytoextraction to roots, stems and leaves of the investigated herbaceous plant species. Significant differences in REEs contents are indicated between plant organs. The highest content was noted in leaves of *P. rhoeas* collected from Area 1 and Area 2 (85.3 ± 8.6 and 50.6 ± 6.0 mg kg^−1^ DW, respectively), roots of ***T. inodorum*** **collected from Area 3 (**71.7 ± 5.1 mg kg^−1^ DW) and leaves of *A. millefolium*, *T. inodorum* and *T. officinale* (38.2 ± 1.1, 44.5 ± 6.4 and 35.8 ± 3.7 mg kg^−1^ DW, respectively) sampled from Area 4.

**Table 3:** Content of total rare earth elements [mg kg^−1^ DW] in organs of plant species growing at experimental areas

Plant species	Plant organ	Area 1	Area 2	Area 3	Area 4
*A. millefolium*	Root	74.4^b^ ± 1.6	8.89^gh^ ± 0.39	27.5^ef^ ± 1.5	31.3^bc^ ± 1.9
	Stem	32.7^efg^ ± 2.1	12.7^fgh^ ± 0.5	29.0^def^ ± 1.7	14.7^efgh^ ± 2.3
	Leaf	44.9^cd^ ± 2.1	36.4^bc^ ± 2.4	24.4^f^ ± 2.2	38.2^ab^ ± 1.1
*A. vulgaris*	Root	15.9^hi^ ± 0.7	10.4^fgh^ ± 0.6	11.1^g^ ± 0.6	8.60^gh^ ± 0.70
	Stem	24.7^gh^ ± 1.0	12.3^fgh^ ± 1.1	11.8^g^ ± 3.8	9.60^fgh^ ± 0.90
	Leaf	37.1^def^ ± 1.1	23.1^de^ ± 2.3	35.7^cde^ ± 3.6	22.9^cde^ ± 3.1
***T. inodorum***	Root	74.4^b^ ± 1.6	29.6^cd^ ± 0.5	71.7^a^ ± 5.1	22.3^de^ ± 1.4
	Stem	32.7^efg^ ± 2.1	22.0^de^ ± 1.0	24.3^f^ ± 4.2	18.2^ef^ ± 0.8
	Leaf	44.9^cd^ ± 2.1	38.1^b^ ± 3.5	38.5^bcd^ ± 1.9	44.5^a^ ± 6.4
*P. rhoeas*	Root	52.4^c^ ± 1.1	17.6^ef^ ± 0.4	46.7^b^ ± 1.2	17.9^ef^ ± 1.5
	Stem	25.3^gh^ ± 2.5	33.2^bc^ ± 1.5	19.0^fg^ ± 1.1	7.20^h^ ± 1.50
	Leaf	85.3^a^ ± 8.6	50.6^a^ ± 6.0	44.5^bc^ ± 3.7	17.3^efg^ ± 1.0
*T. officinale*	Root	10.0^i^ ± 0.6	5.40^h^ ± 0.20	36.9^bcde^ ± 0.8	16.5^efg^ ± 0.3
	Stem	27.9^fg^ ± 1.2	15.3^efg^ ± 0.9	44.6^bc^ ± 1.5	29.7^bcd^ ± 1.8
	Leaf	39.1^de^ ± 3.0	22.0^de^ ± 1.5	44.0^bc^ ± 3.6	35.8^ab^ ± 3.7

*n* = 4, mean values ± SD; identical letters (a, b, c...) followed by values denote no significant (*p* = 0.05) difference in columns according to Tukey’s HSD test (ANOVA)

Moreover, the highest content of REEs was found in the leaves of *A. vulgaris* and *P. rhoeas* in comparison to their content in roots and stems independent of experimental area while the highest content of REEs was recorded in leaves and roots of *A. millefolium* and ***T. inodorum*** **, depending on the place of collection (Table** 3 **).**

#### Content of heavy rare earth elements

Two-way analysis of variance revealed the highly significant (α ≤ 0.001) effect of plant species and plant organs of almost all analysed HREEs, excluding the effect of plant organs and their interaction with species for Ho in Area 1 and Tb in Area 4 (Table S3 in Supplementary data). Total content of HREEs in plant species collected from the tested areas was significantly diverse (Tables S4- S7 in Supplementary data). In the case of Area 1, the highest content of HREEs **was recorded** in roots and leaves of *P. rhoeas* (29.4 and 9.40 mg kg^−1^ DW, respectively) and ***T. inodorum*** **roots (19.4** mg kg^−1^ **DW). The highest content of HREEs** was observed **in** *P. rhoeas* leaves and ***T. inodorum*** **stems** (27.6 and 8.10 mg kg^−1^ DW, respectively) growing in Area 2 . For both experimental areas, Tb and Dy contents were similar to the limits of detection of these elements. Similar findings were recorded for Ho, with the exception of all *A. vulgaris* organs collected from Area 1, where the content of this element was about 0.04 mg kg^−1^ DW. For the remaining HREEs, the highest content of Er, Tm, Y and Sc was noted in ***T. inodorum*** **roots (18.1, 0.26, 0.75 and 0.19** mg kg^−1^ **DW, respectively) and** *P. rhoeas* roots (28.7, 0.11, 0.45 and 0.11 mg kg^−1^ **DW, respectively**) growing in Area 1. In the case of plants collected from Area 2, the highest content of these elements were found in ***T. inodorum*** **stems (7.50, 0.11, 0.27 and 0.11** mg kg^−1^ **DW, respectively) and** *P. rhoeas* leaves (26.5, 0.22, 0.65 and 0.18 mg kg^−1^ **DW, respectively**).

The content of HREEs in plants collected from Areas 3 and 4 was more diverse than in plants from the two previous areas. In the case of Area 3, the highest content of total HREEs was noted in *P. rhoeas* and ***T. inodorum*** roots (17.7 and 9.96 mg kg^−1^ DW, respectively), while from Area 4 in *P. rhoeas* and *A. millefolium* roots (6.78 and 5.65 mg kg^−1^ DW, respectively). Content of Dy and Tb was similar to their limits of detection with the exception of all *P. rhoeas* organs (Dy) growing in Area 4 and also all organs of *P. rhoeas* and *T. officinale* from Area 3 and *P. rhoeas* from Area 4, where their content was significantly higher in comparison to other values. In the case of the remaining elements, the highest amount of Er, Tm, Y and Sc was observed in *P. rhoeas* roots from Area 3 (15.6, 0.26, 1.27 and 0.22 mg kg^−1^ DW, respectively) and Area 4 (5.99, 0.10, 0.49 and 0.09 mg kg^−1^ DW, respectively). It is also worth underlining, that *A. millefolium* collected from Area 4 was characterized by the highest content of Er in its roots and leaves, Y in roots and Sc in stems.

#### Content of light rare earth elements

Two-way ANOVA revealed the highly significant (α ≤ 0.001) effect of plant species and organs on the level of all analysed LREEs in all experimental areas (Table S4 in Supplementary data). Diversity in LREEs was observed in all places where plant species were collected (Tables S8- S11 in Supplementary data). The highest content of total LREEs was recorded in leaves of *A. millefolium* and *P. rhoeas* (75.0 and 76.0 mg kg^−1^ DW, respectively) and *T. inodorum* roots (55.0 mg kg^−1^ DW) collected from Area 1. In the case of Area 2, the highest total content of LREEs in was found *T. inodorum* and *A. millefolium* leaves (37.3 and 34.2 mg kg^−1^ DW) and *P. rhoeas* stems (29.6 mg kg^−1^ DW). Generally, for plants collected from Area 1, the highest content of Gd, Ce, Sm, La and Eu was observed in *T. inodorum* roots, while in *A. millefolium* and *P. rhoeas* leaves the content of Nd was the highest. In the case of Pr, the highest content of this element was in *A. vulgaris* leaves and roots (1.55 and 1.43 mg kg^−1^ DW, respectively) and *T. inodorum* leaves (1.60 mg kg^−1^ DW), and the highest content of Eu was present in *A. vulgaris* leaves and ***T.*** *inodorum s*tems (0.08 and 0.07 mg kg^−1^ DW, respectively). The content of Sm was significantly higher in *T. inodorum* and ***T.*** *officinale* organs than in those of the other plant species.

In the case of plants growing in Area 2, the highest content of Gd, Ce and La was observed in *P. rhoeas* leaves (0.32, 7.10 and 1.26 mg kg^−1^ DW, respectively) and Sm in *T. inodorum* leaves (0.14 mg kg^−1^ DW). *A. millefolium* and *T. inodorum* leaves contained the highest amount of **Nd** (31.2 and 31.1 mg kg^−1^ DW)**,** while Pr was present in the greatest **amount** in *A. vulgaris* leaves and *P. rhoeas* stems (1.35 and 1.20 mg kg^−1^ DW). A similar and the highest content of Eu was recorded in *A. vulgaris* and *T. inodorum* stems and *P. rhoeas* leaves (0.07, 0.08 and 0.07 mg kg^−1^ DW, respectively).

For total LREEs the highest content were present in ***T. inodorum*** **roots,** *T. officinale* leaves and stems and *P. rhoeas* leaves (61.8, 41.4, 37.8 and 37.0 mg kg^−1^ DW, respectively) collected from Area 3, while in plants from Area 4, the highest contents were found in *T. inodorum*, *T. officinale* and *A. millefolium* leaves (43.8, 33.8 and 33.6 mg kg^−1^ DW, respectively). In the case of Area 3, the highest content of Gd and Ce was stated in *T. inodorum*, *P. rhoeas* and *T. officinale* roots. These two plant species were additionally found to have accumulated the most La species, while the highest content of Nd was observed in *T. inodorum* roots (54.7 mg kg^−1^ DW). It is worth noting that a significantly higher content of Sm was stated in *A. millefolium* stems (0.15 mg kg^−1^ DW) and *P. rhoeas* leaves (0.10 mg kg^−1^ DW). Analysis of plants collected from Area 4 revealed a high content of Ce in the majority of the tested plants, especially in their roots and leaves. A significantly higher content of Nd was observed in *T. inodorum*, *A. millefolium* and *T. officinale* leaves (38.3, 27.2 and 27.5 mg kg^−1^ DW, respectively). It is interesting to note that the highest and a similar content of La was stated in *A. millefolium* and *P. rhoeas* roots (0.61 and 0.63 mg kg^−1^ DW, respectively). Additionally, a significantly higher content of Gd and Sm (0.21 and 0.11 mg kg^−1^ DW, respectively) in *P. rhoeas* roots and *A. millefolium* leaves in relation to the rest of the tested plants was observed.

### Similarities and differences between plants from particular areas

**Fig. 2:**
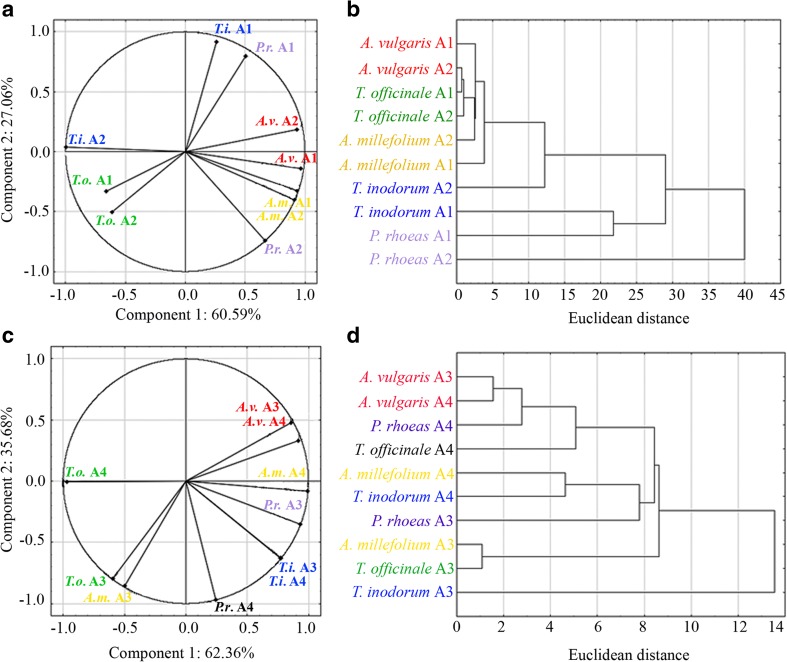
Principal component analysis (PCA) and cluster analysis of HREEs contents in analysed plant species collected from Area 1 and 2 (a and b) and Area 3 and 4 (c and d)

The content of the PCA revealed a positive relation between HREEs contents in *T. inodorum* A1 and *P. rhoeas* A1. Another group with a positive relation was as follows: *A. vulgaris* A2, *A. vulgaris* A1, *A. millefolium* A1, *A. millefolium* A2 and *P. rhoeas* A2. The following species revealed positive relations between them: *T. inodorum* A2, *T. officinale* A1 and *T. officinale* A2, while showing a negative relation with the rest of the tested species and areas (Fig. 2a). Cluster analysis revealed similarities between such groups as *A. vulgaris* A2, *A. vulgaris* A1, *T. officinale* A1, *T. officinale* A2, *A. millefolium* A1 and another group *T. inodorum* A1 and *P. rhoeas* A1 (Fig. 2b). PCA revealed positive relations between all examined species in Areas 3 and 4, excluding *T. officinale* A4, *T. officinale* A3, *A millefolium* A3, which were characterized by positive relations between them and negative to the remaining species and areas. Additionally a strong positive relationship was noted for *A. vulgaris* A3 and *A. vulgaris* A4, as well as for *T. inodorum* A4 and *T. inodorum* A3 (Fig. 2c). Based on the obtained cluster analysis the following groups can be identified: *A. vulgaris* A3, *A. vulgaris* A4, *P. rhoeas* A4, *T. officinale* A4. The next: *A. millefolium* A4 and *T. inodorum* A4 and the last *P. rhoeas* A3, *A. millefolium* A3, *T. officinale* A3, *T. inodorum* A3 (Fig. 2d).

**Fig. 3:**
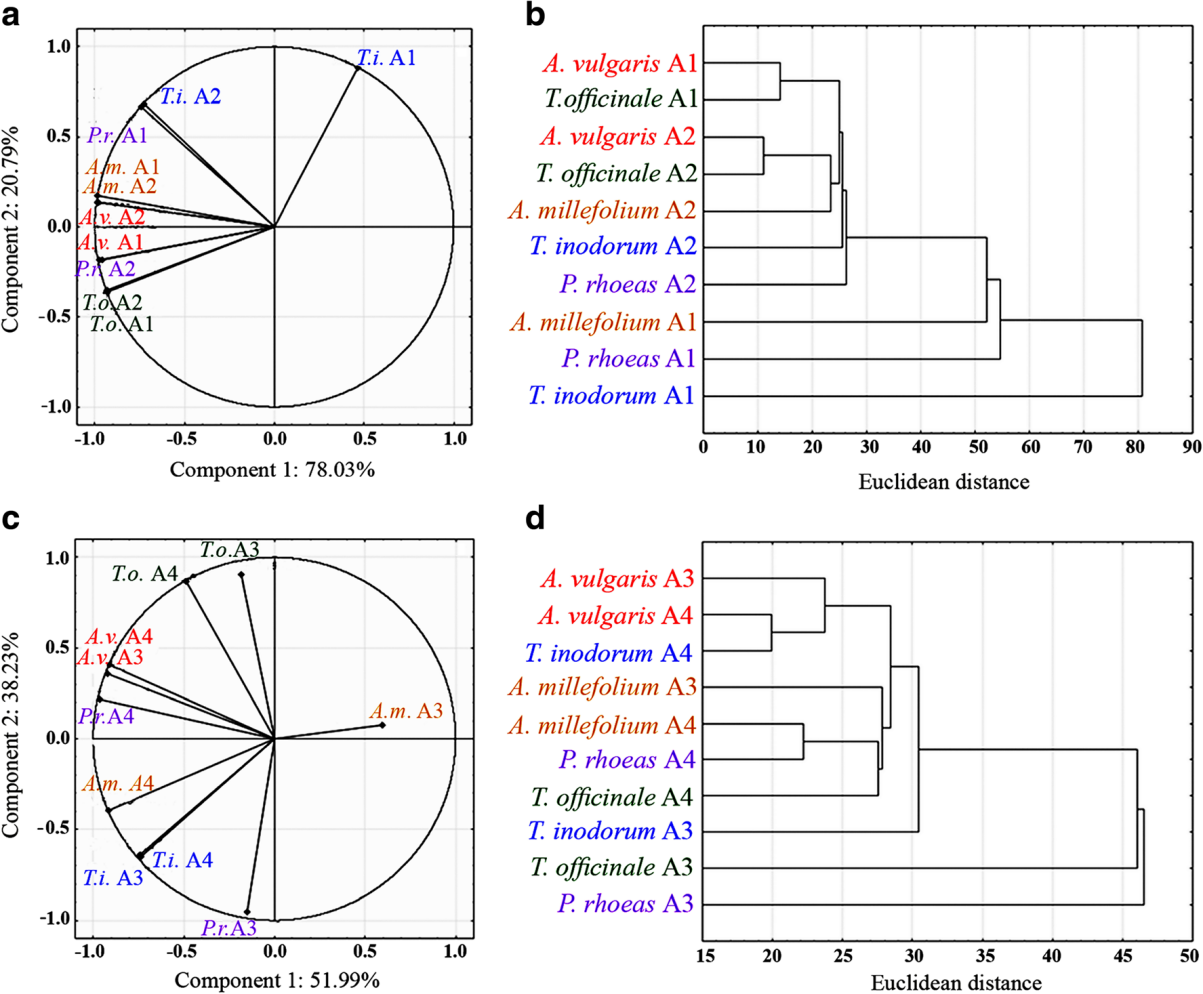
Principal component analysis (PCA) and cluster analysis of REEs contents in analysed plant species collected from Area 1 and 2 (a and b) and Area 3 and 4 (c and d)

For REEs positive relations for Area 1 and Area 2 was observed for all investigated species and areas, excluding *T. inodorum* A1., which was found in a negative relation to *A. millefolium* A1, *A. millefolium* A2, *A. vulgaris* A2, *P. rhoeas* A2, *A. vulgaris* A1, *T. officinale* A2, *T. officinale* A1. Strong positive relations were found between the following pairs of species and areas: *A. millefolium* A1 and *A. millefolium* A2, *T. officinale* A2 and *T. officinale* A1, *P. rhoeas* A2 and *A. vulgaris* A1 (Fig. 3a). Based on cluster analysis three groups were created: *A. vulgaris* A1 with *T. officinale* A1, then *A. vulgaris* A2, *T. officinale* A2, *A. millefolium* A2, *T. inodorum* A2, *P. rhoeas* A2 and the last group *A. millefolium* A1, *P. rhoeas*, *T. inodorum* A1 (Fig. 3b). PCA revealed a negative or a lack of relations between *A. millefolium* A3 with the other experimental objects. A strong positive relation was noted for *A. vulgaris* A4, *A vulgaris* A3 and *P. rhoeas* A4, as well as between *A. millefolium.*, *T. inodorum* A3and *T. inodorum* A4 (Fig. 3c). Cluster analysis allowed three groups with similar responses to be detected: *A. vulgaris* A3, *A. vulgaris* A4, *T. inodorum* A4, the next *A. millefolium* A3, *A. millefolium* A4, *P. rhoeas* A4, *T. officinale* A4. The last group would include *T. officinale* A3 and *P. rhoeas* A3 (Fig. 3d).

**Fig. 4:**
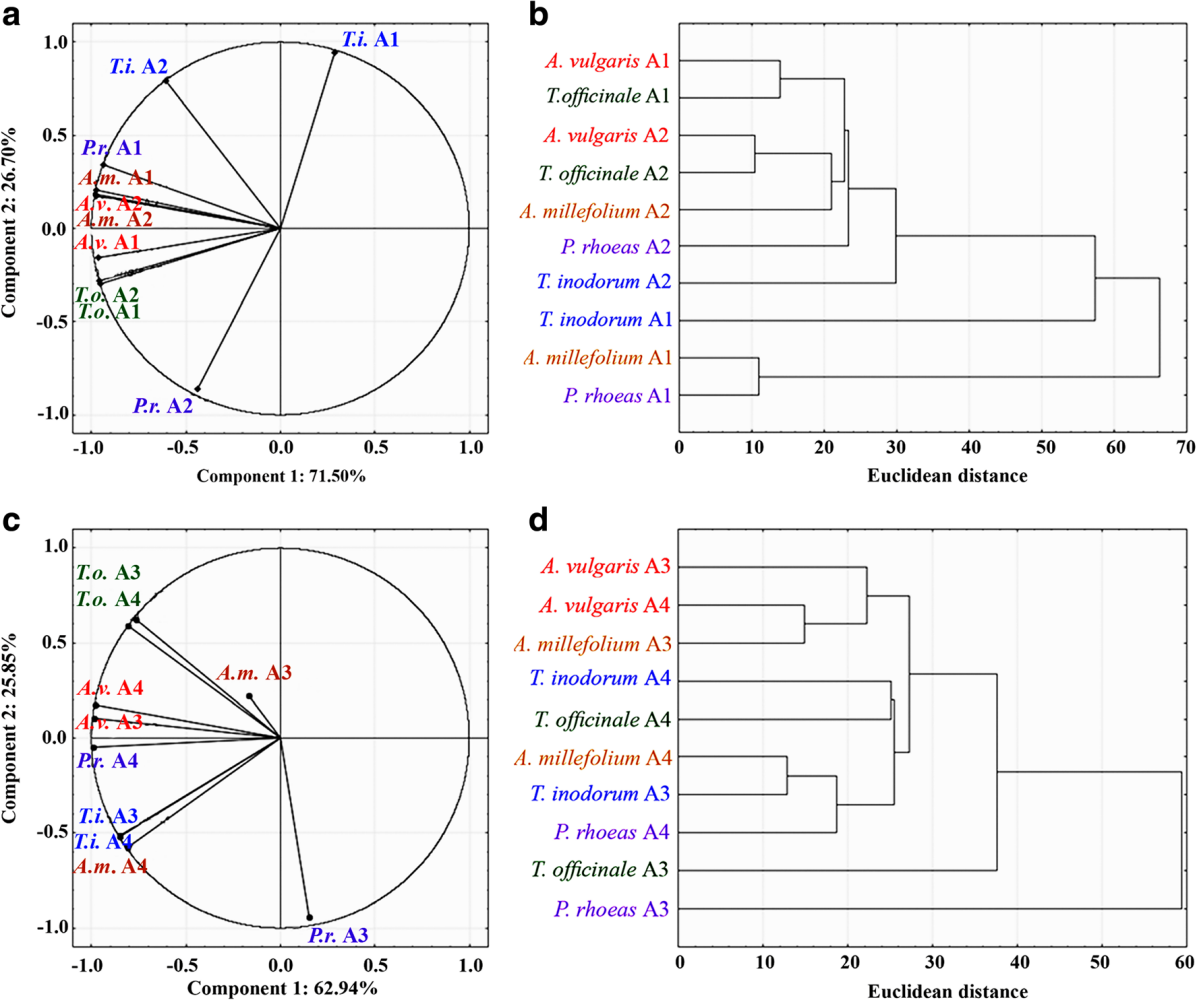
Principal component analysis (PCA) and cluster analysis of LREEs contents in analysed plant species collected from Area 1 and 2 (a and b) and Area 3 and 4 (c and d)

The PCA for LREEs revealed negative relations between *T. inodorum* A1, *T. inodorum* A2 and *P. rhoeas* A2. Simultaneously, *P. rhoeas* A2 was found to lack any relation to other species and areas. A strong positive relation was noted between *A. millefolium* A1 and *A. millefolium* A2 as well as between *T. officinale* A2 and *T. officinale* A1 (Fig. 4a). Cluster analysis revealed the following groups: *A. vulgaris* A1 with *T. officinale* A, next *A. vulgaris* A2, *T. officinale* A2, *A. millefolium* A2, *P. rhoeas* A2, *T. inodorum* A2, and the last, *T. inodorum* A1, *A. millefolium* A1, *P. rhoeas* A1 (Fig. 4b). A negative or a lack of relation was found for *P. rhoeas* A3 with the rest of the experimental objects.

A strong positive relation was noted between *T. officinale* A3 and *T. officinale* A4 as well as between *A. vulgaris* A4 and *A. vulgaris* A3, and between *T. inodorum* A3 and *T. inodorum* A4 (Fig. 4c). Based on cluster analysis the following groups were created: *A. vulgaris* A3, *A. vulgaris* A4, *T. inodorum* A4, then *A. millefolium* A4, *T. officinale* A4. The next group *A. millefolium* A4, *T. inodorum* A3, *P. rhoeas* A4, as well as *T. officinale* A3 and *P. rhoeas* A3 (Fig. 4d).

The PCA of *A. vulgaris* revealed a negative or lack of relation between leaves A1 and roots A2 with other plant organs and areas. A positive relation was found for stems A1, roots A1 and leaves A2 (Fig. S1a in Supplementary data). A positive relation was also recorded for stems A3, leaves A4, roots A3, leaves A3 and stems A4. Roots A4 did not reveal any relation with the other objects (Fig. S1b). *A. millefolium* was found to lack or to show a negative relation of roots A1 with other organs and areas, while the rest revealed positive relations between them (Fig. S2a).

PCA revealed positive relations between plant organs in Areas 3 and 4. A strongly positive relation was found for stems A4 and stems A3 as well as for roots A4 and leaves A4 (Fig. S2b). PCA of REE content in *T. odoratum* revealed positive relations between all plant organs and areas, excluding roots A2, which was found without relations to stems A2 (Fig. S3a). The rest of the species displayed positive relations between plant organs and areas (Fig. S3b; S4a,b; S5a,b).

### Content of REEs, their concentration in soils and road traffic intensity

The efficiency of REEs (HREEs and LREEs) phytoextraction was diverse, therefore for all tested plants, bioconcentration factor (BCF) and translocation factor (TF) values were calculated to show how effective the uptake and transport of these elements were in the plants (Table 4). BCF values were over 1 for all plants collected from particular experimental areas with the exception of almost all plant species collected from Area 2, 3 and 4 and also: *A. vulgaris* from Areas 3 and 4 (HREEs, BCF = 0.87 and 0.63; LREEs, BCF = 0.94 and 0.68, respectively), *A. millefolium* from Area 3 (HREEs, BCF = 0.98), and also *P. rhoeas* from Area 4 (HREEs, BCF = 0.47; LREEs, BCF = 0.52).

**Table 4:** Characteristics of bioaccumulaton (BAF) and translocation (TF) factors with characteristics of two correlations: amount of motor vehicles – concentration of REEs in soil and REEs concentration in soil to their content in plant, calculated based on all observations from all experimental areas (A1-A4)

Plant species	Factor/ correlation	HREEs				LREEs				REEs			
		A1	A2	A3	A4	A1	A2	A3	A4	A1	A2	A3	A4
*A. vulgaris*	BCF	1.90	1.01	0.87	0.63	2.12	1.19	0.94	0.68	1.00	0.55	0.45	0.33
	TF	1.2	1.0	1.4	1.1	2.5	2.3	3.5	2.9	2.3	2.2	3.2	2.7
	Plant - soil	*r* = −0.245, *p* = 0.755				*r* = −0.408, *p* = 0.539				*r* = −0.415, *p* = 0.585			
*A. millefolium*	BCF	2.39	1.40	0.98	1.02	2.66	1.66	1.05	1.11	1.26	0.76	0.51	0.53
	TF	1.6	1.8	0.5	0.8	4.1	4.5	1.0	1.3	3.7	4.1	1.0	1.2
	Plant - soil	*r* = 0.849, *p* = 0.151				*r* = −0.390, *p* = 0.610				*r* = −0.270, *p* = 0.730			
*T. inodorum*	BCF	2.32	1.72	1.15	1.21	2.49	2.03	1.24	1.32	1.19	0.93	0.60	0.63
	TF	0.1	0.3	0.3	0.3	0.8	1.4	1.3	1.3	0.6	1.3	1.0	1.0
	Plant - soil	*r* = 0.275, *p* = 0.725				*r* = −0.612, *p* = 0.388				*r* = −0.525, *p* = 0.475			
*P. rhoeas*	BCF	3.40	2.40	1.17	0.47	3.79	2.83	1.25	0.52	1.79	1.30	0.60	0.25
	TF	0.3	7.8	0.6	0.3	0.8	1.4	0.5	2.2	1.6	2.9	0.5	2.0
	Plant - soil	*r* = −0.889, *p* = 0.111				*r* = −0.056, *p* = 0.944				*r* = −0.348, *p* = 0.652			
*T. officinale*	BCF	2.06	1.07	1.63	1.26	2.30	1.26	1.75	1.38	1.09	0.58	0.84	0.66
	TF	1.0	1.0	0.5	1.4	4.2	4.3	1.3	2.2	3.9	4.1	1.2	2.2
	Plant - soil	*r* = 0.898, *p* = 0.102				*r* = 0.753, *p* = 0.247				*r* = 0.774, *p* = 0.226			
Motor vehicles amount - soil		*r* = 0.992, *p* = 0.008				*r* = 0.996, *p* = 0.004				*r* = 0.995, *p* = 0.005			

For this reason, generally obtained results showed an accumulation of REEs. What is especially interesting is that TF values for the majority plant species (especially *A. vulgaris*, *A. millefolium* and *T. officinale*) growing in particular experimental areas was higher than 1, which suggests the effective phytoextraction and transport of REEs from the root system to leaves. Phytoextraction of REEs with a high ability in the majority plants to translocation of these elements was finally compared with plant/soil correlation values. The values presented in Table 4 showed that for *T. officinale* (LREEs, HREEs and REEs), *A. millefolium* and *T. inodorum* (HREEs) values were significantly positive, whereas negative correlations were found for the rest of the species. It is worth underlining that there were clear correlations between total amount of motor vehicles and concentration of REEs, HREEs and LREEs, which pointed to the significant role of traffic intensity in the transport of these elements to the environment.

### Comparison of the two years of herbaceous plant studies

Analysis of plant material collected in 2015 and 2016 revealed that the above mentioned relationships between plants as regards their ability for total REE phytoextraction and translocation to particular organs (root, stem, leaf) were the same. Moreover, the same relationships between plants collected from particular experimental areas were also identical. However, the efficiency of all tested plant species to phytoextraction of LREEs and HREEs in both years was markedly different. Generally, with three insignificant exceptions, the efficiency of LREEs, HREEs and REEs was higher in 2016 than 2015. The content of total REEs in roots, stems and leaves for all tested plant species was 7–37, 8–42 and 10–44%, respectively; higher in 2016 than 2015. The presentation of the 2015 results only was not accidental as regards to much data of show in regular paper but mainly to confirm the similarities of the relations between plants in particular years.

## Discussion

There are abundant data in the literature that describe the phytoextraction of heavy metals in plants growing in the vicinity of roads (Djingova et al. 2003; Vince et al. 2014; Adelasoye and Alamu 2016), especially as regards the implications for human health (Zhang et al. 2012). Unfortunately, in the case of REEs these data are rather modest, most likely as a result of the analytical difficulties associated with analysis of this element group (Borovička et al. 2011). Although there are currently appropriate tools for REEs analysis our knowledge of the potential of the herbaceous plant species tested in this paper for the phytoextraction of these and other elements is still limited (Keane et al. 2001; Figueiredo et al. 2009; Alvarenga et al. 2015). Djingova et al. (2003) have analysed Ce, La and Nb content in selected plant species and mushrooms collected along streets and highways in Germany. *T. officinale* tested in our paper was found to be one of the most effective in phytoextraction in soil polluted with the analysed elements and heavy metals (Petrova et al. 2013), particularly in its stems and roots (Bini et al. 2012) but also in leaves (Keane et al. 2001). The higher ability of this plant species when compare to the other plants e.g. *A. millefolium* was presented by Gonçalves et al. (2013) as regards selected REEs phytoextraction or by Grzegorczyk et al. (2013) for potassium (K), calcium (Ca) and magnesium (Mg) accumulation. The observed differences, that pointed to a higher or lower content of particular elements in *T. officinale* and *A. millefolium* (Radulescu et al. 2013), cannot indicate the real potential of particular plant species for REEs phytoextraction in their organs. This confirms the results of macroelement content as presented by Ştef et al. (2010) in relation to the data of Grzegorczyk et al. (2013). The same situation, relating to a diverse content of As, Cd, Cu, Pb and Zn in particular *T. officinale* organs, was shown by Bech et al. (2016). Additionally, authors have calculated TF values for the above mentioned metals/metalloids as being generally below 2.1, which reflects the results for total REEs as presented in this paper. It is worth pointing out that the content of HREEs was generally similar or higher in roots than in leaves, whereas in the case of LREEs, the content in leaves was higher than in roots, which confirm the results obtained by Durães et al. (2014). This would suggest that the tested plant species have a limited ability to translocate HREEs from their root system to the aerial parts. This fact is verified by the majority of observations presented using TF values in Table 4.

When we compare the following ratios: stems/roots, leaves/roots and leaves/stems according to data presented by Durães et al. (2014), we did not generally observe any similarities in the differences between these ratios and higher values of ratios, especially for Nd, Gd and Er. Additionally, when comparing BCF calculated for ratios: plant/rhizosphere or organ/rhizosphere, only some relationships were observed. Durães et al. (2014) pointed to differences in the BCF values of particular HREEs and similar values of BCF for LREEs. In our studies only some observations for selected REEs were compared. The reason for the presented differences was probably connected to the higher efficiency of Nd, Er or Ce phytoextraction in the tested plants as well as differences in REEs concentration in soils from particular experimental areas. Environmental factors significantly influence element phytoextraction, as confirmed in numerous papers (Zhuang et al. 2007; Mleczek et al. 2009; Laghlimi et al. 2015). Cao et al. (2001) have shown that changes of pH and redox potential (Eh), affected REEs phytoextraction, especially in relation to Ce, Gd, La and Y. In our study, some relations confirmed this result but for selected elements and tested areas only, which suggests that phytoextraction of REEs is dependent on many other environmental factors.

Ghimire (2015) in her Master’s Thesis described a correlation between the number of vehicles and Cu, Cr, Fe, Ni, Pb and Sn concentration in soil to show generally lower R^2^ values than in our paper. This fact might suggest that one of the sources of REEs is traffic. It is well known that REEs are present not only in electric and hybrid vehicles (eg. La or Dy in NIMH batteries) but also in traditional vehicles (catalytic converters made of Ce, Zr and La or UV cut glass made of Ce) (Lyubomirova et al. 2011). An increase in the amount of REEs used in the production of new vehicles is the reason for the rise of these elements in soil and finally in plants growing near roads. For this reason it is necessary to carefully monitor these elements, perhaps in accordance with the proposals of Angelone et al. (2002), who suggested that the use of platinum (Pt) in catalytic converters be subject to close control.

As data in the literature is significantly limited, to discuss the obtained results we compared the observed relationships describing heavy metal contents presented in other papers. Degórski and Zawiska (2015) have clearly shown that a relationship exists between traffic intensity and Ni, Pb, Cu, Zn, Cd, and Cr concentration in soils, providing confirmation of the major role of this source of heavy metals in the environment. The same observations were documented by Aslam et al. (2013), who found a relationship between the amount of vehicles and Cd, Pb, Cu, Ni, Fe, Mn and Zn concentration in the soil of Dubai (United Arab Emirates). The authors stated that vehicles are the main source of these elements in soil, which in many cases did not correspond to data presented by Oludare et al. (2013) and especially data described by Akpan and William (2014), who have shown Pearson’s correlation in both positive and negative values for these and many other elements. The same observations confirmed the results obtained for REEs (significantly high values of correlation coefficient for amount of motor vehicles and REEs concentration in soil) in this paper, as do those of Czaja et al. (2014) for heavy metals. Our results indicated that the principal source of REEs (both LREEs and HREEs) in soil in the vicinity of roads is motor transport.

The analysis of two years of studies and comparison of the obtained results showed that plants growing in the same experimental areas react in the same way to environmental factors (rain, soil properties or traffic intensity). This was confirmed by the fact that the same relationships were observed between the efficiency of REE phytoextraction in the organs of tested plant species in both 2015 and 2016. Simultaneously, higher phytoextraction of REEs in 2016 was most likely caused by differing weather conditions - drought in 2015, higher temperature, lower precipitation and the standard weather conditions in summer 2016 with more rainy days (Table S2). The obtained results clearly illustrate that the various abilities of the tested plant species for REEs phytoextraction are characteristic of them and independent of the year of growth.

## Conclusions

The problem of toxic elements is especially serious when considering the amount of this environmental pollution and that it results from diverse human activities. New technologies offer numerous promising materials but their production is often related to the emission of various elements directly to the environment. In many cases, REEs remain after the production of new materials and their amount in the environment has markedly increased within the last 15 years. For this reason, biological methods such as phytoremediation should be used to decrease REEs transport within the environment and from entering the food chain. The results presented in this paper have shown the potential of *A. vulgaris, P. rhoeas*, *T. inodorum* and *T. officinale to decontaminate soil in which these elements occur in spite of their clear exclusion being probably effect of REEs immobilization (to high pH value of soil). On the other hand, easy transport of REEs from the* root system to leaves is one of the most important criteria in effective phytoextraction strategy and it has been shown that the above mentioned plant species are able to effectively store REEs in their aboveground plant parts (easy removal of plant parts from environment).

## Electronic supplementary material

Fig S1 (GIF 28 kb)

High resolution (TIFF 1399 kb)

Fig S2 (GIF 28 kb)

High resolution (TIFF 1406 kb)

Fig S3 (GIF 26 kb)

High resolution (TIFF 1367 kb)

Fig S4 (GIF 26 kb)

High resolution (TIFF 1373 kb)

Fig S5 (GIF 27 kb)

High resolution (TIFF 1547 kb)

Table S1 (DOCX 16 kb)

Table S2 (DOCX 15 kb)

Table S3 (DOCX 15 kb)

Table S4 (DOCX 14 kb)

Table S5 (DOCX 17 kb)

Table S6 (DOCX 18 kb)

Table S7 (DOCX 18 kb)

Table S8 (DOCX 18 kb)

Table S9 (DOCX 17 kb)

Table S10 (DOCX 20 kb)

Table S11 (DOCX 20 kb)

Table S12 (DOCX 21 kb)
