# Supplementary figures and images for: Phytoextraction of rare earth elements in herbaceous plant species growing close to roads
Source: Environ Sci Pollut Res Int. 2017 Apr 14;24(16):14091–103. doi: 10.1007/s11356-017-8944-2 (PMC5486614; doi:10.1007/s11356-017-8944-2)

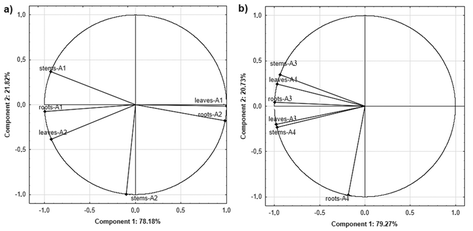

Supplement: Supplementary file 1 — (GIF 28 kb) [file 11356_2017_8944_Fig6_ESM.gif]

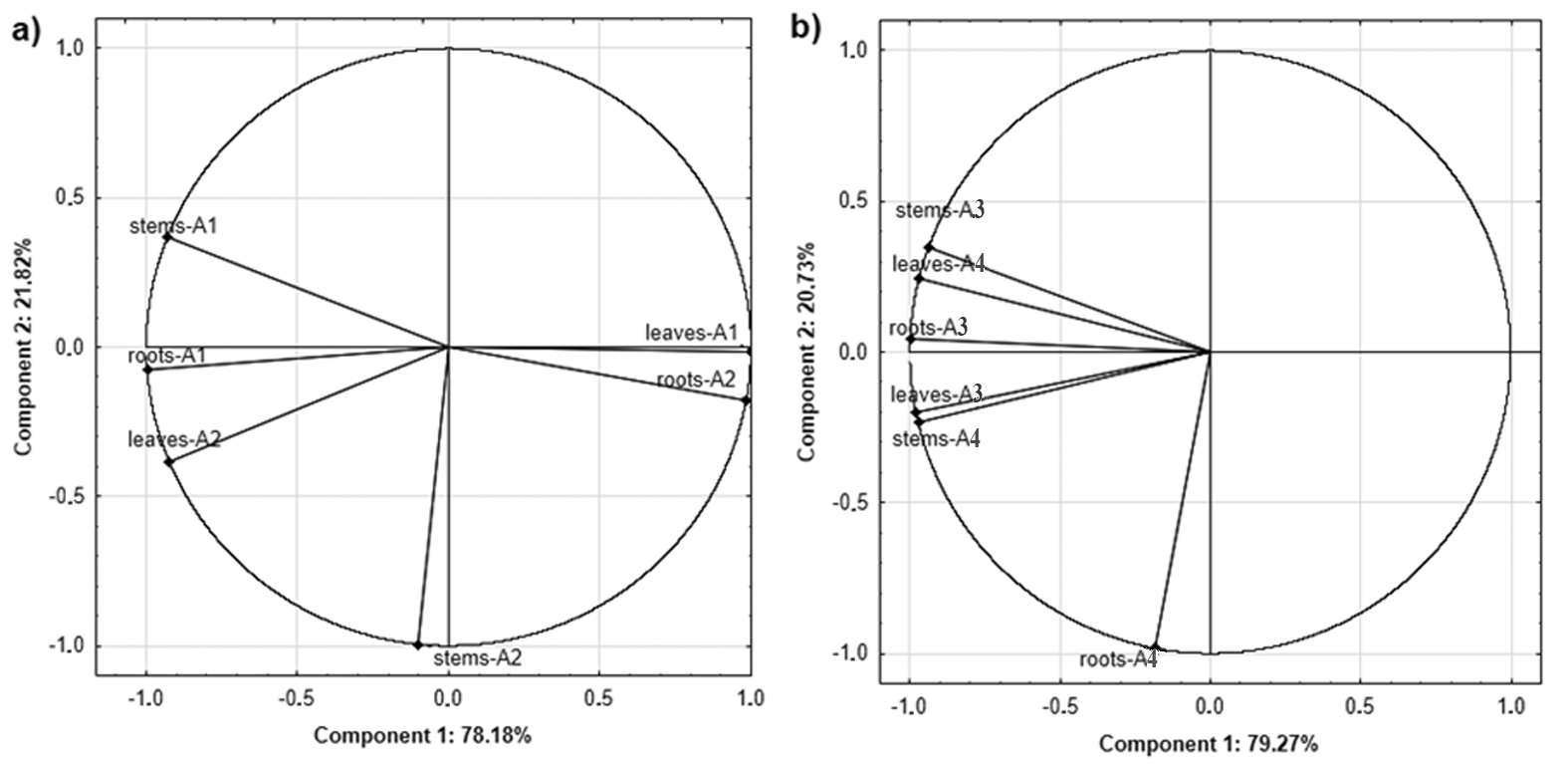

Supplement: Supplementary file 2 — High resolution (TIFF 1399 kb) [file 11356_2017_8944_MOESM1_ESM.tif]

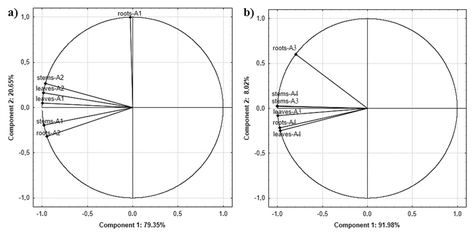

Supplement: Supplementary file 3 — (GIF 28 kb) [file 11356_2017_8944_Fig7_ESM.gif]

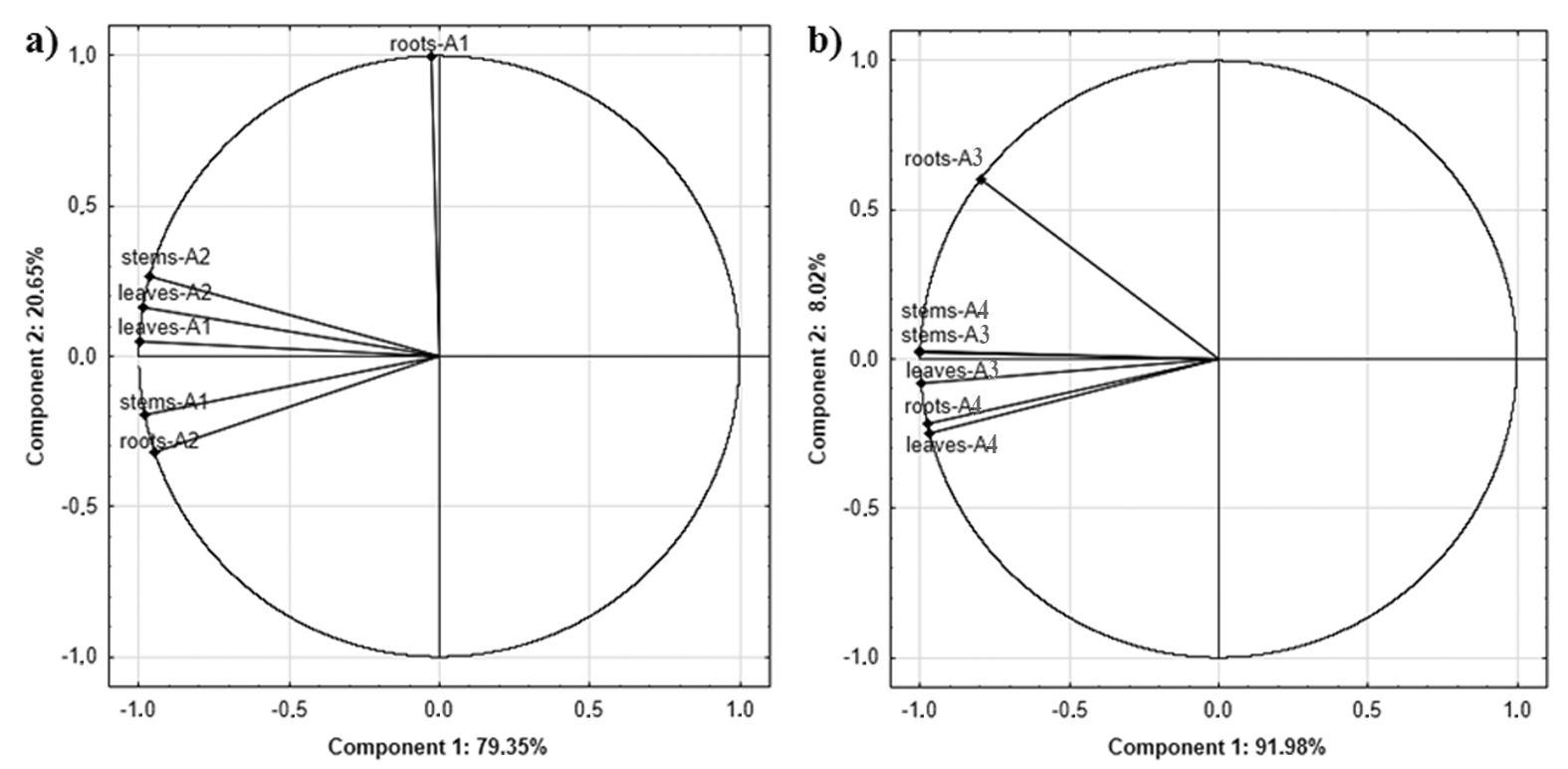

Supplement: Supplementary file 4 — High resolution (TIFF 1406 kb) [file 11356_2017_8944_MOESM2_ESM.tif]

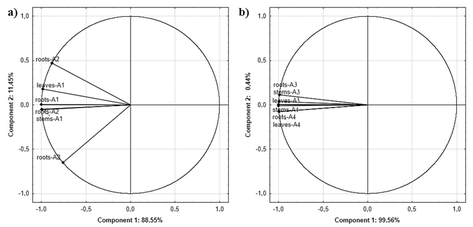

Supplement: Supplementary file 5 — (GIF 26 kb) [file 11356_2017_8944_Fig8_ESM.gif]

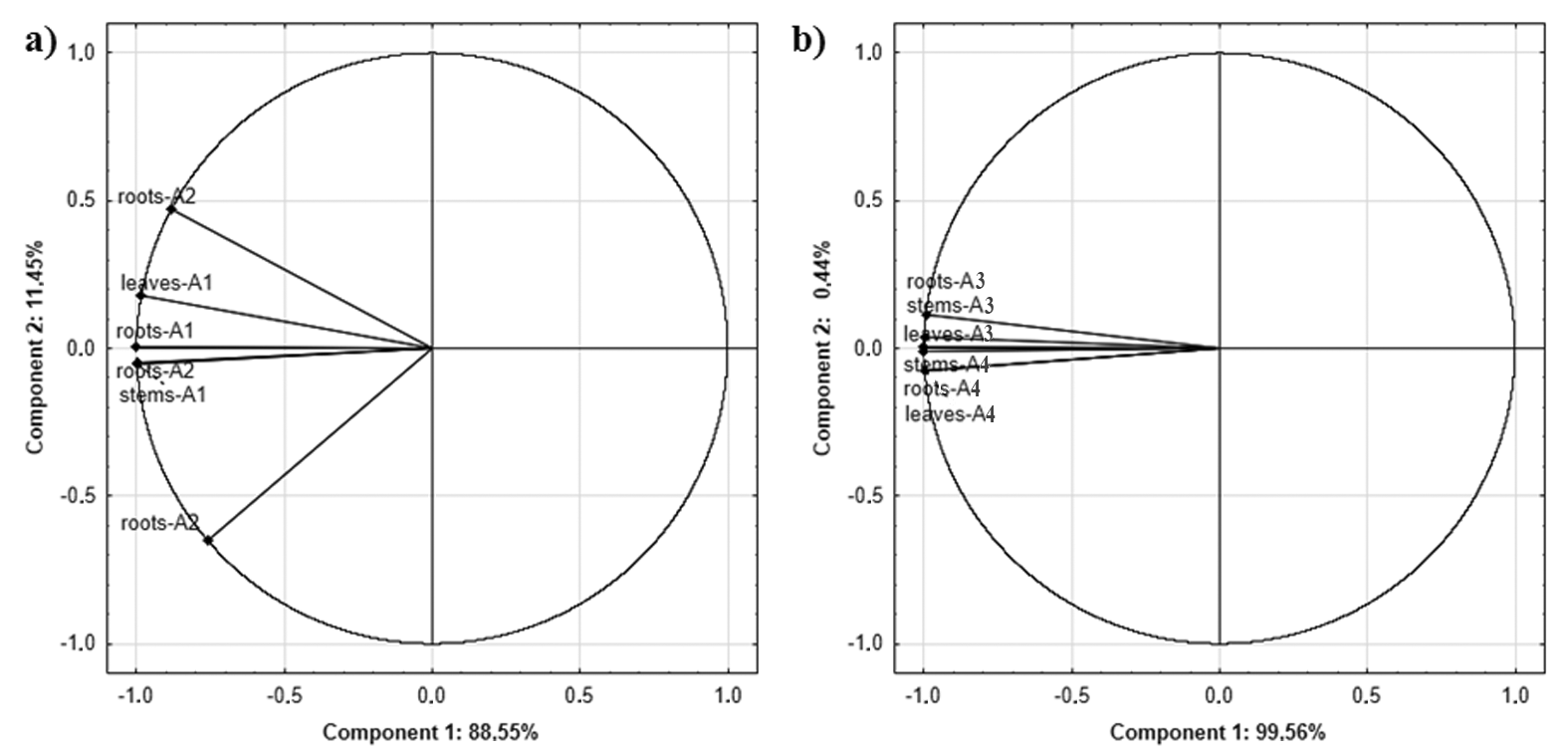

Supplement: Supplementary file 6 — High resolution (TIFF 1367 kb) [file 11356_2017_8944_MOESM3_ESM.tif]

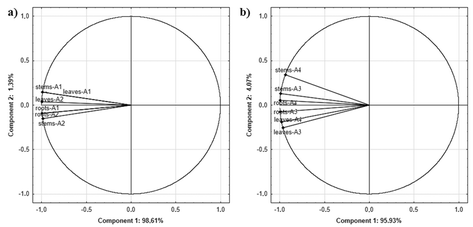

Supplement: Supplementary file 7 — (GIF 26 kb) [file 11356_2017_8944_Fig9_ESM.gif]

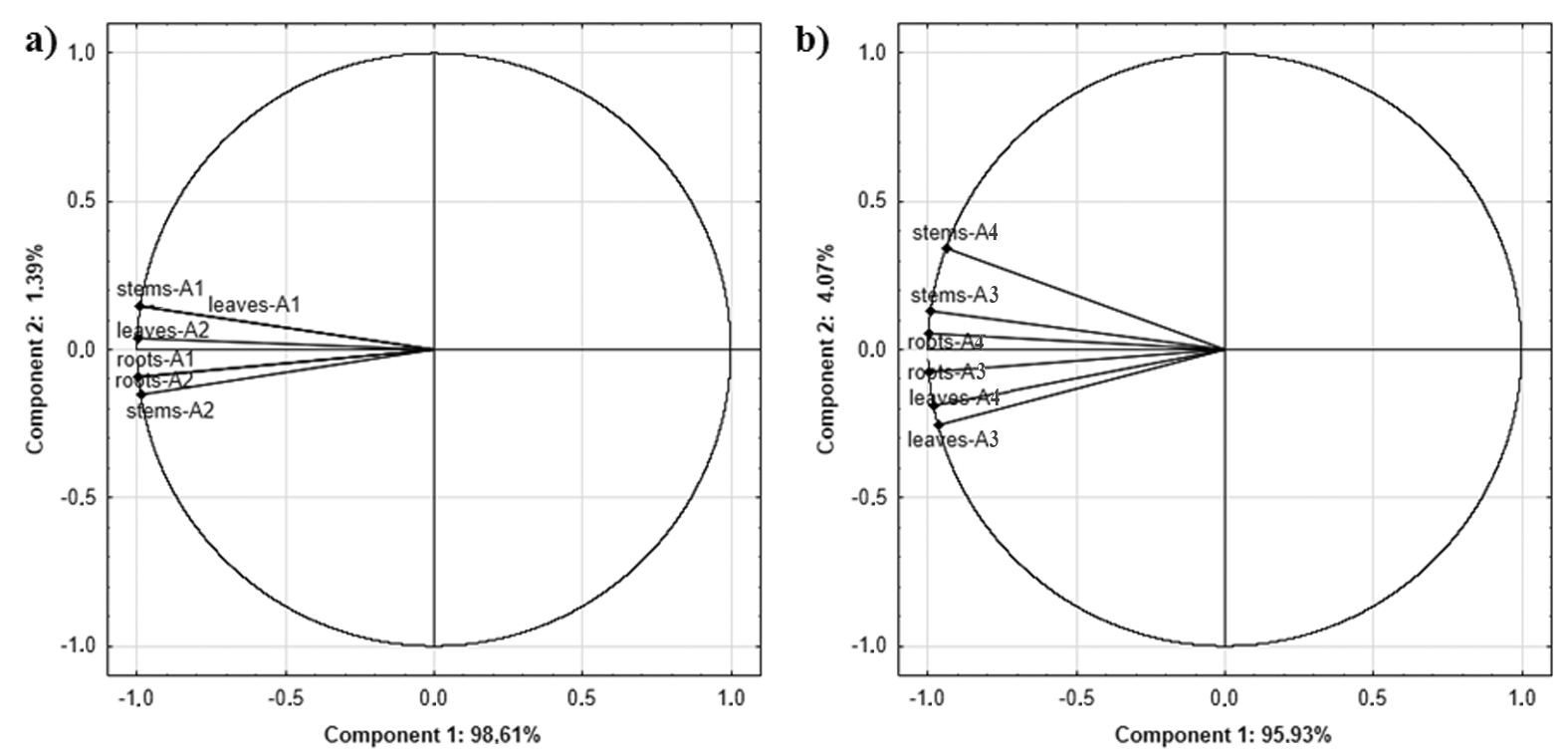

Supplement: Supplementary file 8 — High resolution (TIFF 1373 kb) [file 11356_2017_8944_MOESM4_ESM.tif]

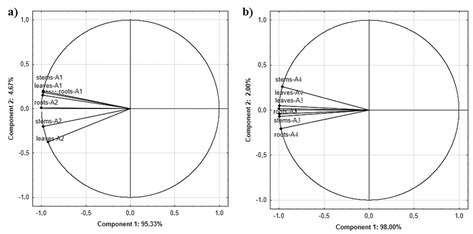

Supplement: Supplementary file 9 — (GIF 27 kb) [file 11356_2017_8944_Fig10_ESM.gif]

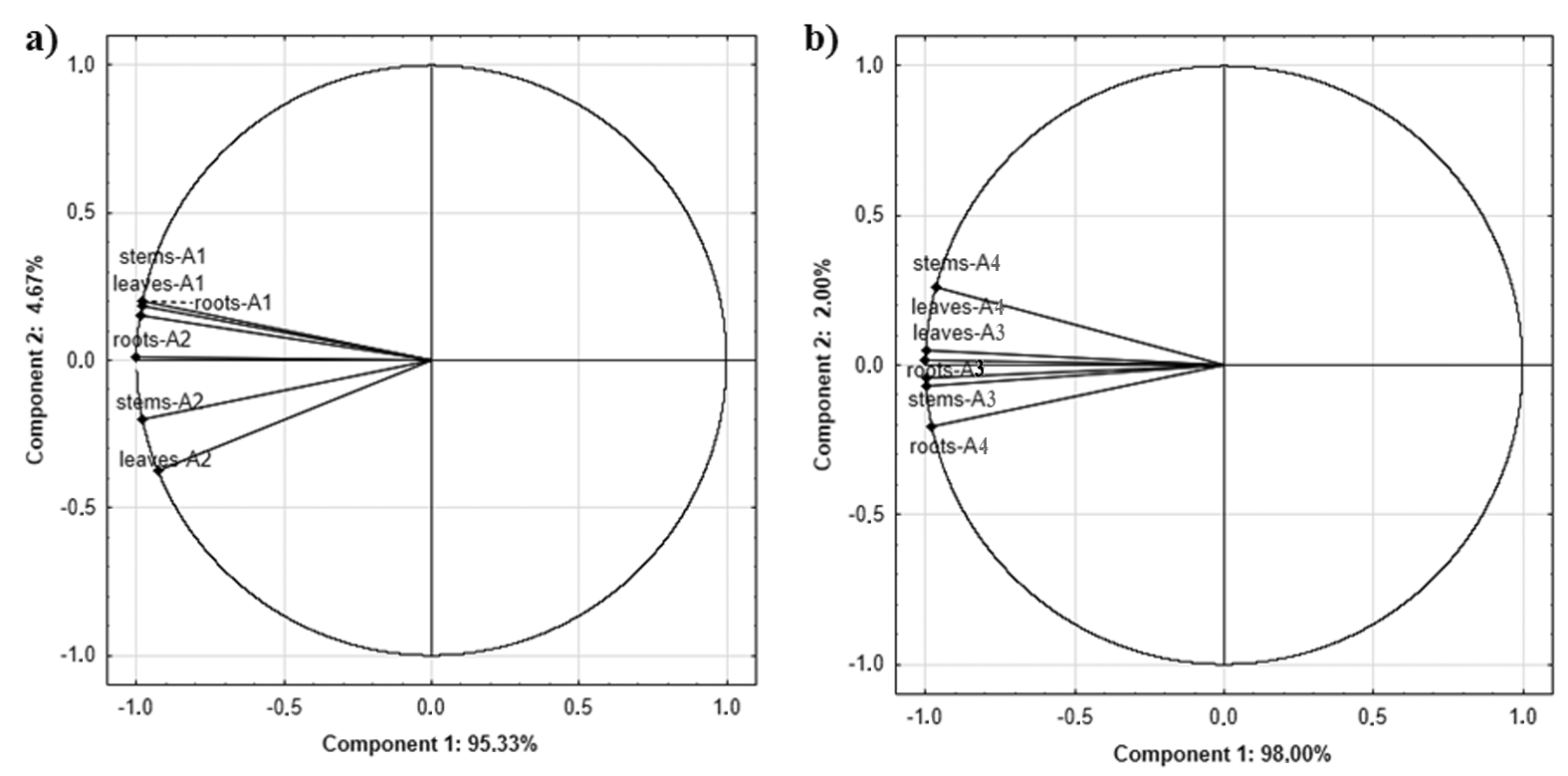

Supplement: Supplementary file 10 — High resolution (TIFF 1547 kb) [file 11356_2017_8944_MOESM5_ESM.tif]
