# Supplementary material for: Phytoextraction of rare earth elements in herbaceous plant species growing close to roads
Source: Environ Sci Pollut Res Int. 2017 Apr 14;24(16):14091–103. doi: 10.1007/s11356-017-8944-2 (PMC5486614; doi:10.1007/s11356-017-8944-2)
Supplement: Supplementary file 11 — (DOCX 16 kb) [file 11356_2017_8944_MOESM6_ESM.docx]

Table S1. Concentration of particular rare earth elements in different components of environment

| HREEs | Air  (pg m^-3^) | Soils  (mg kg^-1^) | | Plants  (µg kg^-1^) |
| --- | --- | --- | --- | --- |
| Gd | 1 | 0.7 – 5 | | vegetables < 2; grass 35;  pine needles 25 |
| Ce | South Pole. 0.8 – 5*;  Spitsbergen 20 – 60*;  Great Britain 330 – 550* | 2 – 75 | | vegetables 2 – 50;  grass 330;  pine needles 370 |
| Sm | South Pole 0.03 – 0.1;  Great Britain 20 – 40;  USA 70 - 1000 | 0.2 – 6 | | vegetables 0.2 – 100;  grass 53;  pine needles 32 |
| La | South Pole 0.2 – 1.4;  Spitsbergen 5 – 30;  USA 500 - 9000 | 1.5 – 40 | | vegetables 0.4 – 2000;  grass 170;  pine needles ~ 300 |
| Nd | ~300 | 1 – 30 | | vegetables 10; grass 150;  pine needles 160 |
| Pr | 60 | 1.5 – 9 | | vegetables 1 – 2; grass 40;  pine needles 60 – 130 |
| Eu | South Pole 0.004 – 0.02; Spitsbergen 1 – 2;  Germany 5 – 80; | < 1 – 1.5 | | vegetables 0.04 – 70;  grass 8;  pine needles 5 |
| LREEs | Air  (pg m^-3^) | Soils  (mg kg^-1^) | | Plants  (µg kg^-1^) |
| Lu | Spitsbergen 0.4 – 3 | 0.05 – 0.45 | | vegetables 0.01 – 60;  grass 3; pine needles 2 |
| Er | 24 | 0.02 – 2 | | vegetables 0.5 – 2;  pine needles 6;  blue berry – leaves 1.5 – 8 |
| Ho | 13 | 0.02 – 1 | | vegetables 0.06 – 0.1;  grass < 20; pine needles 4 – 5 |
| Tb | Greenland 1 – 5;  Garmany 10;  USA 20 – 35 | 0.05 – 1 | | vegetables 0.1 – 2;  grass 9;  pine needles 20 |
| Tm | 4 | 0.03 – 0.05 | | vegetables 0.02 – 4;  grass 50; pine needles 1.5 |
| Y | Atmospheric dust  0.02 – 2 ppm. | World 5 – 24. Poland 5 – 12 | different 0.01 – 3.5;  lichens and briophytes do 200 | |
| Yb | 4 – >10 | 0.07 – 2.5 | vegetables 0.01 – 20;  grass 20; pine needles 8 | |
| Dy | 40 | 1 – 5 | vegetables 20;  blue berry – leaves 21 | |
| Sc | South Pole 0.06 – 0.2; Spitsbergen 0.01 – 5;  USA 80 – 3000 | World: podzolic loam 5; brown 8; mould 10. Poland: sandy soils 2; clay 6 | different 8 – 700; tea 10 – 140; fungi 2 – 300 | |

*- values in ng m^-3^; data in table prepared according to Kabata-Pendias and Pendias (1999)
