# Supplementary material for: Phytoextraction of rare earth elements in herbaceous plant species growing close to roads
Source: Environ Sci Pollut Res Int. 2017 Apr 14;24(16):14091–103. doi: 10.1007/s11356-017-8944-2 (PMC5486614; doi:10.1007/s11356-017-8944-2)
Supplement: Supplementary file 12 — (DOCX 15 kb) [file 11356_2017_8944_MOESM7_ESM.docx]

Table S2. Characteristics of air temperature, air relative humidity and particulate matter concentrations within five months before the samples collection time in 2015 and 2016

| Year | Month | Air temperature  [°C] | | | Air relative humidity [%] | | | Particulate matter [PM10]  [µg m^-3^] | | | Sum of precipitation [mm] |
| --- | --- | --- | --- | --- | --- | --- | --- | --- | --- | --- | --- |
|  |  | Mean | Min | Max | Mean | Min | Max | Mean | Min | Max |  |
| 2015 | IV | 9 | 2 | 17 | 64 | 48 | 92 | 25 | 7 | 65 | 20 |
|  | V | 14 | 9 | 19 | 65 | 53 | 83 | 19 | 10 | 32 | 27 |
|  | VI | 16 | 11 | 24 | 71 | 40 | 93 | 15 | 7 | 29 | 55 |
|  | VII | 20 | 14 | 27 | 66 | 48 | 90 | 17 | 10 | 29 | 51 |
|  | VIII | 23 | 17 | 29 | 58 | 41 | 79 | 27 | 15 | 42 | 25 |
|  | Average | 16 | 11 | 23 | 65 | 46 | 87 | 21 | 10 | 39 | 36 |
| 2016 | IV | 9 | 4 | 17 | 68 | 50 | 90 | 31 | 14 | 83 | 61 |
|  | V | 16 | 8 | 23 | 68 | 49 | 90 | 23 | 12 | 35 | 52 |
|  | VI | 19 | 15 | 27 | 71 | 50 | 93 | 18 | 9 | 38 | 74 |
|  | VII | 19 | 15 | 25 | 75 | 58 | 99 | 16 | 6 | 35 | 134 |
|  | VIII | 18 | 14 | 23 | 76 | 60 | 93 | 17 | 8 | 33 | 48 |
|  | Average | 16 | 11 | 23 | 72 | 53 | 93 | 21 | 10 | 45 | 74 |

Source: http://poznan.wios.gov.pl/, http://www.imgw.pl/klimat/
