# Supplementary material for: Phytoextraction of rare earth elements in herbaceous plant species growing close to roads
Source: Environ Sci Pollut Res Int. 2017 Apr 14;24(16):14091–103. doi: 10.1007/s11356-017-8944-2 (PMC5486614; doi:10.1007/s11356-017-8944-2)
Supplement: Supplementary file 13 — (DOCX 15 kb) [file 11356_2017_8944_MOESM8_ESM.docx]

Table S3. Results of two-way analysis of variance (F test statistics and significance levels) of HREEs with plant species and plant organs fixed factors

| Factor | **Heavy Rare Earth Elements** | | | | | | | | | |
| --- | --- | --- | --- | --- | --- | --- | --- | --- | --- | --- |
|  | **Lu** | **Er** | **Ho** | **Tb** | **Tm** | **Y** | **Yb** | **Dy** | **Sc** | **Total** |
| **Area 1** | | | | | | | | | | |
| species | 577.6*** | 1454.4*** | 3847.4*** | nd | 1254.5*** | 1278.1*** | 16.2*** | nd | 194.6*** | 1380.6*** |
| plant organ | 131.2*** | 3945.9*** | 0.3ns | nd | 3079.0*** | 4244.2*** | 186.7*** | nd | 1404.9*** | 3932.1*** |
| species×organ interaction | 119.2*** | 735.9*** | 0.3ns | nd | 785.6*** | 824.9*** | 87.5*** | nd | 331.2*** | 714.1*** |
| **Area 2** | | | | | | | | | | |
| species | 1082.9*** | 169.3*** | nd | nd | 147.4*** | 146.0*** | 102.7*** | nd | 102.6*** | 168.4*** |
| plant organ | 126.6*** | 78.7*** | nd | nd | 88.0*** | 67.7*** | 13.4*** | nd | 79.7*** | 77.9*** |
| species×organ interaction | 130.2*** | 114.9*** | nd | nd | 114.3*** | 118.8*** | 27.5*** | nd | 98.9*** | 114.6*** |
| **Area 3** | | | | | | | | | | |
| species | 18.7*** | 352.9*** | 18.5*** | 1966.5*** | 175.0*** | 887.2*** | 245.5*** | nd | 245.8*** | 353.9*** |
| plant organ | 12.5*** | 462.5*** | 7.5*** | 85.3*** | 348.6*** | 2524.7*** | 42.9*** | nd | 120.1*** | 491.8*** |
| species×organ interaction | 27.9*** | 218.9*** | 26.6*** | 76.2*** | 118.5*** | 830.7*** | 89.8*** | nd | 239.8*** | 221.1*** |
| **Area 4** | | | | | | | | | | |
| species | 220.5*** | 209.2*** | 245.9*** | 481.1*** | 83.7*** | 160.7*** | 436.5*** | 329.6*** | 104.5*** | 201.8*** |
| plant organ | 22.2*** | 149.5*** | 8.3*** | 0.1ns | 103.6*** | 637.4*** | 69.7*** | 3.7** | 54.6*** | 143.2*** |
| species×organ interaction | 43.2*** | 124.7*** | 24.3*** | 0.1ns | 62.6*** | 147.2*** | 56.1*** | 3.7*** | 64.1*** | 106.1*** |

nd – not detected; ns – not significant; significance levels - *** p<0.001; ** p<0.01; * p<0.05
