# Supplementary material for: Phytoextraction of rare earth elements in herbaceous plant species growing close to roads
Source: Environ Sci Pollut Res Int. 2017 Apr 14;24(16):14091–103. doi: 10.1007/s11356-017-8944-2 (PMC5486614; doi:10.1007/s11356-017-8944-2)
Supplement: Supplementary file 14 — (DOCX 14 kb) [file 11356_2017_8944_MOESM9_ESM.docx]

Table S4. Results of two-way analysis of variance (F test statistics and significance levels) of LREEs with plant species and plant organs fixed factors

| Factor | **Light Rare Earth Elements** | | | | | | | |
| --- | --- | --- | --- | --- | --- | --- | --- | --- |
|  | **Gd** | **Ce** | **Sm** | **La** | **Nd** | **Pr** | **Eu** | **Total** |
| **Area 1** | | | | | | | | |
| species | 759.1*** | 90.3*** | 1235.7*** | 1174.0*** | 89.1*** | 412.3*** | 232.1*** | 91.4*** |
| plant organ | 2807.1*** | 33.9*** | 431.3*** | 3692.6*** | 224.1*** | 171.0*** | 104.6*** | 179.3*** |
| species×organ interaction | 647.6*** | 63.5*** | 75.8*** | 809.2*** | 85.3*** | 25.7*** | 104.1*** | 79.4*** |
| **Area 2** | | | | | | | | |
| species | 126.5*** | 139.7*** | 411.9*** | 175.6*** | 54.1*** | 237.6*** | 150.8*** | 64.9*** |
| plant organ | 79.1*** | 82.2*** | 90.0*** | 47.4*** | 310.3*** | 10.5*** | 75.6*** | 245.6*** |
| species×organ interaction | 96.7*** | 15.7*** | 69.8*** | 99.8*** | 71.4*** | 51.5*** | 88.6*** | 42.4*** |
| **Area 3** | | | | | | | | |
| species | 903.6*** | 67.9*** | 579.9*** | 565.3*** | 94.1*** | 92.5*** | 82.1*** | 78.5*** |
| plant organ | 2579.6*** | 51.4*** | 97.9*** | 1712.8*** | 70.4*** | 26.5*** | 20.2*** | 59.9*** |
| species×organ interaction | 699.4*** | 41.1*** | 217.3*** | 442.9*** | 60.9*** | 84.1*** | 13.1*** | 45.4*** |
| **Area 4** | | | | | | | | |
| species | 189.2*** | 88.5*** | 491.8*** | 168.7*** | 66.4*** | 48.6*** | 101.3*** | 67.2*** |
| plant organ | 519.9*** | 49.3*** | 11.9*** | 472.9*** | 178.6*** | 21.1*** | 65.1*** | 140.5*** |
| species×organ interaction | 168.4*** | 19.8*** | 113.0*** | 123.5*** | 14.9*** | 28.6*** | 42.7*** | 13.6*** |

nd – not detected; ns – not significant; significance levels - *** p<0.001; ** p<0.01; * p<0.05
