# Supplementary material for: Phytoextraction of rare earth elements in herbaceous plant species growing close to roads
Source: Environ Sci Pollut Res Int. 2017 Apr 14;24(16):14091–103. doi: 10.1007/s11356-017-8944-2 (PMC5486614; doi:10.1007/s11356-017-8944-2)
Supplement: Supplementary file 15 — (DOCX 17 kb) [file 11356_2017_8944_MOESM10_ESM.docx]

Table S5. Content of heavy rare earth elements [mg kg^-1^ DW] in plant species growing at Area 1

| Plant species | Plant organ | Lu | Er | Ho | Tb | Tm | Y | Yb | Dy | Sc | Total HRREs |
| --- | --- | --- | --- | --- | --- | --- | --- | --- | --- | --- | --- |
| *A. millefolium* | Root | 0.04^b^ | 2.72^e^ | bDL | bDL | 0.08^c^ | 0.11^f^ | 0.04^b^ | bDL | 0.04^f^ | 3.02^e^ |
|  | Stem | 0.03^b^ | 0.97^f^ | bDL | bDL | 0.04^d^ | 0.04^g^ | 0.04^bc^ | bDL | 0.04^f^ | 1.15^f^ |
|  | Leaf | 0.03^b^ | 4.32^d^ | bDL | bDL | 0.07^c^ | 0.28^c^ | 0.03^bc^ | bDL | 0.07^e^ | 4.81^d^ |
| *A. vulgaris* | Root | 0.10^a^ | 1.87^ef^ | 0.04^b^ | bDL | 0.04^d^ | 0.07^f^ | 0.04^b^ | bDL | 0.13^b^ | 2.30^ef^ |
|  | Stem | 0.04^b^ | 1.02^f^ | 0.04^a^ | bDL | 0.04^d^ | 0.04^g^ | 0.04^b^ | bDL | 0.11^c^ | 1.32^f^ |
|  | Leaf | 0.04^b^ | 2.39^e^ | 0.04^a^ | bDL | 0.04^d^ | 0.19^d^ | 0.04^b^ | bDL | 0.09^d^ | 2.82^e^ |
| ***T. inodorum*** | Root | 0.04^b^ | 18.1^b^ | bDL | bDL | 0.26^a^ | 0.74^a^ | 0.07^a^ | bDL | 0.19^a^ | 19.4^b^ |
|  | Stem | 0.04^b^ | 2.03^ef^ | bDL | bDL | 0.04^d^ | 0.11^f^ | 0.03^c^ | bDL | 0.04^f^ | 2.28^ef^ |
|  | Leaf | 0.04^b^ | 1.45^cd^ | bDL | bDL | 0.04^d^ | 0.15^e^ | 0.04^b^ | bDL | 0.04^f^ | 1.75^f^ |
| ***P. rhoeas*** | Root | 0.04^b^ | 28.7^a^ | bDL | bDL | 0.11^b^ | 0.45^b^ | 0.04^b^ | bDL | 0.11^c^ | 29.4^a^ |
|  | Stem | 0.03^b^ | 2.29^e^ | bDL | bDL | 0.08^c^ | 0.04^g^ | 0.07^a^ | bDL | 0.04^f^ | 2.55^ef^ |
|  | Leaf | 0.03^b^ | 9.04^c^ | bDL | bDL | 0.04^d^ | 0.18^de^ | 0.04^b^ | bDL | 0.04^f^ | 9.37^c^ |
| *T. officinale* | Root | bDL | 0.59^f^ | bDL | bDL | bDL | 0.07^f^ | 0.07^a^ | bDL | 0.04^f^ | 0.78^f^ |
|  | Stem | bDL | 0.70^f^ | bDL | bDL | bDL | 0.04^g^ | 0.04^b^ | bDL | 0.07^e^ | 0.86^f^ |
|  | Leaf | bDL | 0.62^f^ | bDL | bDL | bDL | 0.04^g^ | 0.04^b^ | bDL | 0.07^e^ | 0.78^f^ |

Mean values (n=3) ± SD; identical letters (a, b, c..) followed by values denote no significant (p = 0.05) difference in columns according to Tukey's HSD test (ANOVA)

bDL – below detection limit
