# Supplementary material for: Phytoextraction of rare earth elements in herbaceous plant species growing close to roads
Source: Environ Sci Pollut Res Int. 2017 Apr 14;24(16):14091–103. doi: 10.1007/s11356-017-8944-2 (PMC5486614; doi:10.1007/s11356-017-8944-2)
Supplement: Supplementary file 16 — (DOCX 18 kb) [file 11356_2017_8944_MOESM11_ESM.docx]

Table S6. Content of heavy rare earth elements [mg kg^-1^ DW] in plant species growing at Area 2

| Plant species | Plant organ | Lu | Er | Ho | Tb | Tm | Y | Yb | Dy | Sc | Total HRREs |
| --- | --- | --- | --- | --- | --- | --- | --- | --- | --- | --- | --- |
| *A. millefolium* | Root | 0.02^b^ | 1.12^c^ | bDL | bDL | 0.03^c^ | 0.05^d^ | 0.02^c^ | bDL | 0.02^d^ | 1.25^d^ |
|  | Stem | 0.02^b^ | 0.44^cd^ | bDL | bDL | 0.02^cd^ | 0.02^d^ | 0.02^c^ | bDL | 0.02^d^ | 0.52^d^ |
|  | Leaf | 0.02^b^ | 1.97^c^ | bDL | bDL | 0.03^c^ | 0.13^c^ | 0.02^c^ | bDL | 0.03^c^ | 2.20^cd^ |
| *A. vulgaris* | Root | 0.04^a^ | 0.57^d^ | bDL | bDL | 0.02^cd^ | 0.11^c^ | 0.04^b^ | bDL | 0.04^c^ | 0.82^d^ |
|  | Stem | 0.04^a^ | 0.33^d^ | bDL | bDL | 0.02^cd^ | 0.01^d^ | 0.03^b^ | bDL | 0.03^c^ | 0.43^d^ |
|  | Leaf | 0.04^a^ | 0.69^d^ | bDL | bDL | 0.03^c^ | 0.01^d^ | 0.03^b^ | bDL | 0.03^c^ | 0.80^d^ |
| ***T. inodorum*** | Root | 0.04^a^ | 2.42^cd^ | bDL | bDL | 0.04^c^ | 0.11^c^ | 0.03^b^ | bDL | 0.04^c^ | 2.68^cd^ |
|  | Stem | 0.04^a^ | 7.50^b^ | bDL | bDL | 0.11^b^ | 0.26^b^ | 0.04^b^ | bDL | 0.11^b^ | 8.07^b^ |
|  | Leaf | 0.04^a^ | 0.57^cd^ | bDL | bDL | 0.04^c^ | 0.07^cd^ | 0.04^b^ | bDL | 0.03^c^ | 0.79^d^ |
| ***P. rhoeas*** | Root | 0.04^a^ | 3.29^c^ | bDL | bDL | 0.02^cd^ | 0.11^c^ | 0.04^b^ | bDL | 0.04^c^ | 3.54^c^ |
|  | Stem | 0.04^a^ | 3.33^c^ | bDL | bDL | 0.04^c^ | 0.07^cd^ | 0.04^b^ | bDL | 0.04^c^ | 3.55^c^ |
|  | Leaf | 0.04^a^ | 26.5^a^ | bDL | bDL | 0.22^a^ | 0.65^a^ | 0.07^a^ | bDL | 0.2^a^ | 27.6^a^ |
| *T. officinale* | Root | bDL | 0.32^d^ | bDL | bDL | bDL | 0.04^d^ | 0.04^b^ | bDL | 0.02^d^ | 0.42^d^ |
|  | Stem | bDL | 0.39^d^ | bDL | bDL | bDL | 0.02^d^ | 0.02^c^ | bDL | 0.04^c^ | 0.47^d^ |
|  | Leaf | bDL | 0.35^d^ | bDL | bDL | bDL | 0.02^d^ | 0.02^c^ | bDL | 0.04^c^ | 0.44^d^ |

Mean values (n=3) ± SD; identical letters (a, b, c..) followed by values denote no significant (p = 0.05) difference in columns according to Tukey's HSD test (ANOVA)

bDL – below detection limit
