# Supplementary material for: Phytoextraction of rare earth elements in herbaceous plant species growing close to roads
Source: Environ Sci Pollut Res Int. 2017 Apr 14;24(16):14091–103. doi: 10.1007/s11356-017-8944-2 (PMC5486614; doi:10.1007/s11356-017-8944-2)
Supplement: Supplementary file 17 — (DOCX 18 kb) [file 11356_2017_8944_MOESM12_ESM.docx]

Table S7. Content of heavy rare earth elements [mg kg^-1^ DW] in plant species growing at Area 3

| Plant species | Plant organ | Lu | Er | Ho | Tb | Tm | Y | Yb | Dy | Sc | Total HRREs |
| --- | --- | --- | --- | --- | --- | --- | --- | --- | --- | --- | --- |
| *A. millefolium* | Root | 0.04^a^ | 5.41^d^ | 0.04^b^ | bDL | 0.07^d^ | 0.25^e^ | 0.04^c^ | bDL | 0.07^c^ | 5.91^d^ |
|  | Stem | bDL | 6.47^c^ | 0.07^a^ | bDL | 0.07^d^ | 0.15^ef^ | 0.04^c^ | bDL | 0.04^d^ | 6.85^c^ |
|  | Leaf | 0.04^a^ | 2.23^h^ | 0.04^b^ | bDL | 0.04^e^ | 0.07^g^ | 0.04^c^ | bDL | 0.37^a^ | 2.83^ef^ |
| *A. vulgaris* | Root | 0.04^a^ | 1.46^gh^ | 0.03^b^ | bDL | 0.04^e^ | 0.04^g^ | bDL | bDL | bDL | 1.61^f^ |
|  | Stem | 0.04^a^ | 0.36^h^ | 0.03^b^ | bDL | 0.03^e^ | bDL | bDL | bDL | bDL | 0.46^g^ |
|  | Leaf | 0.04^a^ | 1.94^g^ | 0.04^b^ | bDL | 0.04^e^ | 0.12^f^ | bDL | bDL | bDL | 2.18^f^ |
| ***T. inodorum*** | Root | 0.04^a^ | 9.26^b^ | 0.04^b^ | bDL | 0.15^b^ | 0.37^c^ | 0.04^c^ | bDL | 0.07^c^ | 9.96^b^ |
|  | Stem | 0.04^a^ | 1.86^g^ | 0.04^b^ | bDL | 0.08^d^ | 0.04^g^ | 0.04^c^ | bDL | 0.04^d^ | 2.12^f^ |
|  | Leaf | 0.04^a^ | 5.38^d^ | 0.04^b^ | bDL | 0.11^c^ | 0.18^f^ | 0.04^c^ | bDL | 0.04^d^ | 5.82^d^ |
| ***P. rhoeas*** | Root | 0.04^a^ | 15.6^a^ | 0.07^a^ | 0.04^b^ | 0.26^a^ | 1.23^a^ | 0.11^a^ | bDL | 0.22^b^ | 17.6^a^ |
|  | Stem | 0.04^a^ | 3.17^f^ | 0.04^b^ | 0.04^b^ | 0.04^e^ | 0.07^g^ | 0.04^c^ | bDL | 0.04^d^ | 3.46^e^ |
|  | Leaf | bDL | 5.30^d^ | 0.04^b^ | 0.04^b^ | 0.07^d^ | 0.10^f^ | 0.03^c^ | bDL | 0.04^d^ | 5.61^d^ |
| *T. officinale* | Root | 0.04^a^ | 4.48^e^ | 0.04^b^ | 0.07^a^ | 0.11^c^ | 0.56^b^ | 0.04^c^ | bDL | 0.07^c^ | 5.42^e^ |
|  | Stem | 0.04^a^ | 6.15^cd^ | 0.04^b^ | 0.07^a^ | 0.07^d^ | 0.33^d^ | 0.04^c^ | bDL | 0.07^c^ | 6.82^c^ |
|  | Leaf | 0.04^a^ | 2.23^g^ | 0.04^b^ | 0.04^b^ | 0.04^e^ | 0.11^f^ | 0.07^b^ | bDL | 0.04^d^ | 2.61^f^ |

Mean values (n=3) ± SD; identical letters (a, b, c..) followed by values denote no significant (p = 0.05) difference in columns according to Tukey's HSD test (ANOVA)

bDL – below detection limit

Mean values (n=3) ± SD; identical letters (a, b, c..) followed by values denote no significant (p = 0.05) difference in columns according to Tukey's HSD test (ANOVA)

bDL – below detection limit
