# Supplementary material for: Phytoextraction of rare earth elements in herbaceous plant species growing close to roads
Source: Environ Sci Pollut Res Int. 2017 Apr 14;24(16):14091–103. doi: 10.1007/s11356-017-8944-2 (PMC5486614; doi:10.1007/s11356-017-8944-2)
Supplement: Supplementary file 18 — (DOCX 18 kb) [file 11356_2017_8944_MOESM13_ESM.docx]

Table S8. Content of heavy rare earth elements [mg kg^-1^ DW] in plant species growing at Area 4

| Plant species | Plant organ | Lu | Er | Ho | Tb | Tm | Y | Yb | Dy | Sc | Total HRREs |
| --- | --- | --- | --- | --- | --- | --- | --- | --- | --- | --- | --- |
| *A. millefolium* | Root | 0.04^a^ | 4.92^b^ | 0.04^b^ | bDL | 0.11^a^ | 0.42^b^ | 0.04^b^ | bDL | 0.08^b^ | 5.64^b^ |
|  | Stem | 0.04^a^ | 1.38^f^ | 0.04^b^ | bDL | 0.07^c^ | 0.04^fg^ | 0.04^b^ | bDL | 0.36^a^ | 1.96^e^ |
|  | Leaf | 0.04^a^ | 4.29^c^ | 0.04^b^ | bDL | 0.04^d^ | 0.11^e^ | 0.07^a^ | bDL | 0.04^c^ | 4.63^c^ |
| *A. vulgaris* | Root | 0.03^b^ | 1.13^f^ | bDL | bDL | 0.03^de^ | 0.03^fg^ | bDL | bDL | bDL | 1.23^f^ |
|  | Stem | 0.03^b^ | 0.30^g^ | 0.03^bc^ | bDL | 0.03^de^ | bDL | bDL | bDL | bDL | 0.38^g^ |
|  | Leaf | 0.02^b^ | 1.25^f^ | 0.02^c^ | bDL | 0.02^de^ | 0.07^ef^ | bDL | bDL | bDL | 1.34^f^ |
| ***T. inodorum*** | Root | bDL | 1.94^e^ | 0.07^a^ | bDL | 0.04^d^ | 0.11^e^ | bDL | bDL | 0.04^c^ | 2.21^e^ |
|  | Stem | bDL | 1.26^f^ | 0.04^b^ | bDL | 0.04^d^ | 0.04^fg^ | bDL | bDL | 0.04^c^ | 1.41^f^ |
|  | Leaf | bDL | 0.50^g^ | 0.07^a^ | bDL | 0.04^d^ | 0.07^f^ | bDL | bDL | 0.04^c^ | 0.71^g^ |
| ***P. rhoeas*** | Root | 0.01^c^ | 5.98^a^ | 0.03^bc^ | bDL | 0.10^b^ | 0.48^a^ | 0.04^b^ | 0.029^b^ | 0.09^b^ | 6.78^a^ |
|  | Stem | 0.01^c^ | 1.28^f^ | 0.02^cd^ | bDL | 0.01^e^ | 0.03^fg^ | 0.01^c^ | 0.026^b^ | 0.01^cd^ | 1.43^f^ |
|  | Leaf | bDL | 2.14^e^ | 0.01^d^ | bDL | 0.03^de^ | 0.04^f^ | 0.01^c^ | 0.037^a^ | 0.01^cd^ | 2.30^e^ |
| *T. officinale* | Root | bDL | 1.12^f^ | bDL | bDL | 0.04^d^ | 0.22^c^ | 0.04^b^ | bDL | bDL | 1.42^f^ |
|  | Stem | 0.04^a^ | 3.58^d^ | bDL | bDL | 0.08^c^ | 0.15^d^ | 0.04^b^ | bDL | 0.04^c^ | 3.93^d^ |
|  | Leaf | 0.04^a^ | 1.68^ef^ | bDL | bDL | 0.04^d^ | 0.11^e^ | 0.07^a^ | bDL | 0.04^c^ | 1.98^ef^ |

Mean values (n=3) ± SD; identical letters (a, b, c..) followed by values denote no significant (p = 0.05) difference in columns according to Tukey's HSD test (ANOVA)

bDL – below detection limit

Mean values (n=3) ± SD; identical letters (a, b, c..) followed by values denote no significant (p = 0.05) difference in columns according to Tukey's HSD test (ANOVA)

bDL – below detection limit
