# Supplementary material for: Phytoextraction of rare earth elements in herbaceous plant species growing close to roads
Source: Environ Sci Pollut Res Int. 2017 Apr 14;24(16):14091–103. doi: 10.1007/s11356-017-8944-2 (PMC5486614; doi:10.1007/s11356-017-8944-2)
Supplement: Supplementary file 19 — (DOCX 17 kb) [file 11356_2017_8944_MOESM14_ESM.docx]

Table S9. Content of light rare earth elements [mg kg^-1^ DW] in plant species growing at Area 1

| Plant species | Plant organ | Gd | Ce | Sm | La | Nd | Pr | Eu | Total LREEs |
| --- | --- | --- | --- | --- | --- | --- | --- | --- | --- |
| *A. millefolium* | Root | 0.04^d^ | 4.05^d^ | bDL | 0.27^ef^ | 12.9^e^ | 1.18^b^ | 0.04^b^ | 18.5^de^ |
|  | Stem | 0.07^d^ | 4.84^cd^ | bDL | 0.16^g^ | 21.0^de^ | 0.68^d^ | 0.04^b^ | 26.8^d^ |
|  | Leaf | 0.10^c^ | 5.65^b^ | bDL | 0.49^c^ | 68.4^a^ | 0.28^f^ | 0.04^b^ | 75.0^a^ |
| *A. vulgaris* | Root | 0.04^e^ | 3.09^e^ | bDL | 0.22^f^ | 8.82^e^ | 1.43^a^ | 0.04^b^ | 13.6^e^ |
|  | Stem | 0.04^d^ | 4.00^d^ | bDL | 0.11^g^ | 18.1^de^ | 1.14^bc^ | 0.04^b^ | 23.4^d^ |
|  | Leaf | 0.04^d^ | 5.34^b^ | bDL | 0.38^d^ | 26.9^cd^ | 1.55^a^ | 0.08^a^ | 34.3^cd^ |
| ***T. inodorum*** | Root | 0.33^a^ | 8.61^a^ | 0.07^a^ | 1.52^a^ | 43.1^b^ | 1.26^b^ | 0.07^a^ | 54.9^b^ |
|  | Stem | 0.04^d^ | 5.08^bc^ | 0.04^c^ | 0.25^f^ | 23.9^d^ | 1.09^c^ | 0.07^a^ | 30.4^d^ |
|  | Leaf | 0.04^d^ | 5.46^bc^ | 0.04^c^ | 0.30^cd^ | 35.7^bc^ | 1.60^a^ | 0.04^b^ | 43.2^c^ |
| ***P. rhoeas*** | Root | 0.19^b^ | 5.94^b^ | bDL | 0.67^b^ | 15.5^de^ | 0.67^d^ | 0.04^b^ | 23.0^d^ |
|  | Stem | 0.09^d^ | 4.29^d^ | bDL | 0.11^e^ | 18.2^de^ | 0.04^g^ | 0.04^b^ | 22.7^d^ |
|  | Leaf | 0.11^c^ | 6.22^b^ | bDL | 0.32^e^ | 68.5^a^ | 0.86^d^ | 0.02^c^ | 76.0^a^ |
| *T. officinale* | Root | 0.04^d^ | 2.35^e^ | 0.07^a^ | 0.18^fg^ | 6.10^e^ | 0.44^e^ | 0.04^b^ | 9.2^e^ |
|  | Stem | 0.04^d^ | 5.03^c^ | 0.05^b^ | 0.15^g^ | 21.0^de^ | 0.67^d^ | 0.04^b^ | 27.0^d^ |
|  | Leaf | 0.02^e^ | 4.96^c^ | 0.04^c^ | 0.11^g^ | 32.6^c^ | 0.59^d^ | 0.02^c^ | 38.3^c^ |

Mean values (n=3) ± SD; identical letters (a, b, c..) followed by values denote no significant (p = 0.05) difference in columns according to Tukey's HSD test (ANOVA)

bDL – below detection limit

Mean values (n=3) ± SD; identical letters (a, b, c..) followed by values denote no significant (p = 0.05) difference in columns according to Tukey's HSD test (ANOVA)

bDL – below detection limit

Mean values (n=3) ± SD; identical letters (a, b, c..) followed by values denote no significant (p = 0.05) difference in columns according to Tukey's HSD test (ANOVA)

bDL – below detection limit
