# Supplementary material for: Phytoextraction of rare earth elements in herbaceous plant species growing close to roads
Source: Environ Sci Pollut Res Int. 2017 Apr 14;24(16):14091–103. doi: 10.1007/s11356-017-8944-2 (PMC5486614; doi:10.1007/s11356-017-8944-2)
Supplement: Supplementary file 20 — (DOCX 20 kb) [file 11356_2017_8944_MOESM15_ESM.docx]

Table S10. Content of light rare earth elements [mg kg^-1^ DW] in plant species growing at Area 2

| Plant species | Plant organ | Gd | Ce | Sm | La | Nd | Pr | Eu | Total LREEs |
| --- | --- | --- | --- | --- | --- | --- | --- | --- | --- |
| *A. millefolium* | Root | 0.02^d^ | 1.67^e^ | bDL | 0.11^de^ | 5.33^de^ | 0.49^d^ | 0.02^c^ | 7.64^e^ |
|  | Stem | 0.03^cd^ | 2.20^de^ | bDL | 0.06^e^ | 9.54^d^ | 0.31^e^ | 0.02^c^ | 12.2^d^ |
|  | Leaf | 0.05^cd^ | 2.58^d^ | bDL | 0.22^d^ | 31.2^a^ | 0.13^f^ | 0.02^c^ | 34.2^a^ |
| *A. vulgaris* | Root | 0.04^d^ | 1.59^e^ | bDL | 0.15^de^ | 6.93^e^ | 0.83^cd^ | 0.04^b^ | 9.58^de^ |
|  | Stem | 0.10^b^ | 1.94^de^ | bDL | 0.07^e^ | 8.98^de^ | 0.77^d^ | 0.07^a^ | 11.9^d^ |
|  | Leaf | 0.03^d^ | 3.14^cd^ | bDL | 0.07^e^ | 17.7^c^ | 1.35^a^ | 0.03^b^ | 22.3^c^ |
| ***T. inodorum*** | Root | 0.04^d^ | 4.67^bc^ | 0.04^c^ | 0.37^c^ | 20.9^b^ | 0.92^b^ | 0.04^b^ | 26.9^bc^ |
|  | Stem | 0.11^b^ | 3.86^c^ | 0.08^b^ | 0.57^b^ | 8.26^d^ | 0.98^b^ | 0.08^a^ | 13.9^d^ |
|  | Leaf | 0.07^c^ | 4.97^b^ | 0.14^a^ | 0.18^cde^ | 31.1^a^ | 0.89^b^ | bDL | 37.3^a^ |
| ***P. rhoeas*** | Root | 0.07^c^ | 2.91^d^ | 0.04^c^ | 0.26^d^ | 9.94^d^ | 0.78^c^ | 0.04^b^ | 14.0^d^ |
|  | Stem | 0.04^d^ | 5.23^b^ | 0.04^c^ | 0.26^d^ | 22.8^b^ | 1.20^a^ | 0.04^b^ | 29.6^b^ |
|  | Leaf | 0.32^a^ | 7.09^a^ | 0.07^b^ | 1.26^a^ | 13.8^c^ | 0.40^e^ | 0.07^a^ | 23.0^c^ |
| *T. officinale* | Root | 0.02^d^ | 1.28^e^ | 0.04^c^ | 0.10^e^ | 3.32^e^ | 0.24^ef^ | 0.02^c^ | 5.02^e^ |
|  | Stem | 0.02^d^ | 2.76^d^ | 0.03^cd^ | 0.08^e^ | 11.6^cd^ | 0.37^e^ | 0.02^c^ | 14.8^d^ |
|  | Leaf | 0.01^d^ | 2.80^d^ | 0.02^d^ | 0.06^e^ | 18.4^b^ | 0.33^e^ | 0.01^c^ | 21.6^c^ |

Mean values (n=3) ± SD; identical letters (a, b, c..) followed by values denote no significant (p = 0.05) difference in columns according to Tukey's HSD test (ANOVA)

bDL – below detection limit
