# Supplementary material for: Phytoextraction of rare earth elements in herbaceous plant species growing close to roads
Source: Environ Sci Pollut Res Int. 2017 Apr 14;24(16):14091–103. doi: 10.1007/s11356-017-8944-2 (PMC5486614; doi:10.1007/s11356-017-8944-2)
Supplement: Supplementary file 21 — (DOCX 20 kb) [file 11356_2017_8944_MOESM16_ESM.docx]

Table S11. Content of light rare earth elements [mg kg^-1^ DW] in plant species growing at Area 3

| Plant species | Plant organ | Gd | Ce | Sm | La | Nd | Pr | Eu | Total LREEs |
| --- | --- | --- | --- | --- | --- | --- | --- | --- | --- |
| *A. millefolium* | Root | 0.11^d^ | 4.66^c^ | 0.04^d^ | 0.46^e^ | 14.9^ef^ | 1.42^a^ | 0.04^b^ | 21.6^d^ |
|  | Stem | 0.07^e^ | 4.48^c^ | 0.15^a^ | 0.30^g^ | 16.2^e^ | 0.89^c^ | 0.04^b^ | 22.2^d^ |
|  | Leaf | bDL | 4.47^c^ | 0.04^d^ | 0.19^hi^ | 16.7^e^ | 0.11^f^ | 0.04^b^ | 21.5^d^ |
| *A. vulgaris* | Root | bDL | 1.57^e^ | bDL | 0.07^j^ | 6.45^f^ | 1.35^a^ | 0.04^b^ | 9.49^f^ |
|  | Stem | bDL | 1.81^e^ | bDL | 0.07^j^ | 8.84^ef^ | 0.56^d^ | 0.03^b^ | 11.3^ef^ |
|  | Leaf | bDL | 4.78^c^ | bDL | 0.19^hi^ | 27.6^c^ | 0.97^bc^ | 0.04^b^ | 33.5^bc^ |
| ***T. inodorum*** | Root | 0.22^b^ | 5.94^bc^ | bDL | 0.63^c^ | 54.7^a^ | 0.18^f^ | 0.07^a^ | 61.8^a^ |
|  | Stem | 0.04^f^ | 4.24^d^ | bDL | 0.15^i^ | 17.6^e^ | bDL | 0.08^a^ | 22.1^d^ |
|  | Leaf | 0.07^e^ | 5.05^c^ | bDL | 0.37^f^ | 26.3^c^ | 0.84^c^ | 0.07^a^ | 32.7^bc^ |
| ***P. rhoeas*** | Root | 0.56^a^ | 9.05^a^ | 0.04^d^ | 1.64^a^ | 16.9^e^ | 0.78^c^ | 0.07^a^ | 29.0^c^ |
|  | Stem | 0.04^f^ | 2.57^d^ | 0.04^d^ | 0.15^i^ | 11.4^ef^ | 1.27^ab^ | 0.04^b^ | 15.5^e^ |
|  | Leaf | 0.04^f^ | 4.25^c^ | 0.10^b^ | 0.21^h^ | 31.3^bc^ | 1.09^b^ | 0.04^b^ | 37.0^b^ |
| *T. officinale* | Root | 0.22^b^ | 6.73^b^ | 0.07^c^ | 1.08^b^ | 22.5^d^ | 0.78^c^ | 0.04^b^ | 31.5^c^ |
|  | Stem | 0.15^c^ | 6.30^bc^ | 0.07^c^ | 0.56^d^ | 29.8^c^ | 0.89^c^ | 0.04^b^ | 37.8^b^ |
|  | Leaf | 0.04^f^ | 5.18^c^ | 0.04^d^ | 0.22^h^ | 35.5^b^ | 0.37^e^ | bDL | 41.4^b^ |

Mean values (n=3) ± SD; identical letters (a, b, c..) followed by values denote no significant (p = 0.05) difference in columns according to Tukey's HSD test (ANOVA)

bDL – below detection limit
