# Supplementary material for: Phytoextraction of rare earth elements in herbaceous plant species growing close to roads
Source: Environ Sci Pollut Res Int. 2017 Apr 14;24(16):14091–103. doi: 10.1007/s11356-017-8944-2 (PMC5486614; doi:10.1007/s11356-017-8944-2)
Supplement: Supplementary file 22 — (DOCX 21 kb) [file 11356_2017_8944_MOESM17_ESM.docx]

**Supplementary data**

Table S12. Content of light rare earth elements [mg kg^-1^ DW] in plant species growing at Area 4

| *A. millefolium* | Plant organ | Gd | Ce | Sm | La | Nd | Pr | Eu | Total LREEs |
| --- | --- | --- | --- | --- | --- | --- | --- | --- | --- |
| *A. vulgaris* | Root | 0.15^b^ | 5.38^a^ | 0.08^b^ | 0.61^a^ | 18.4^c^ | 1.02^b^ | 0.04^b^ | 25.6^c^ |
|  | Stem | 0.04^d^ | 2.46^c^ | 0.04^c^ | 0.04^f^ | 9.17^de^ | 0.94^b^ | 0.04^b^ | 12.7^d^ |
|  | Leaf | 0.04^d^ | 4.93^a^ | 0.11^a^ | 0.26^d^ | 27.2^b^ | 0.98^b^ | 0.07^a^ | 33.6^b^ |
| ***T. inodorum*** | Root | bDL | 1.22^d^ | bDL | 0.06^f^ | 5.00^e^ | 1.05^b^ | 0.03^bc^ | 7.36^e^ |
|  | Stem | bDL | 1.48^d^ | bDL | 0.05^f^ | 7.24^de^ | 0.46^cd^ | 0.03^c^ | 9.26^cd^ |
|  | Leaf | bDL | 3.07^bc^ | bDL | 0.12^e^ | 17.7^c^ | 0.62^c^ | 0.02^c^ | 21.5^cd^ |
| ***P. rhoeas*** | Root | 0.04^d^ | 3.62^b^ | 0.07^b^ | 0.19^e^ | 15.5^d^ | 0.67^c^ | 0.04^b^ | 20.1^cd^ |
|  | Stem | 0.04^d^ | 2.90^bc^ | 0.08^b^ | 0.08^f^ | 12.8^cd^ | 0.88^b^ | 0.04^b^ | 16.8^cd^ |
|  | Leaf | 0.04^d^ | 4.65^a^ | 0.04^c^ | 0.14^e^ | 38.3^a^ | 0.60^c^ | 0.04^b^ | 43.8^a^ |
| *T. officinale* | Root | 0.21^a^ | 3.46^b^ | 0.01^d^ | 0.63^a^ | 6.47^de^ | 0.30^d^ | 0.03^bc^ | 11.1^de^ |
|  | Stem | 0.02^e^ | 1.00^d^ | 0.02^d^ | 0.07^f^ | 4.73^e^ | 0.54^c^ | 0.01^d^ | 6.39^e^ |
|  | Leaf | 0.01^ef^ | 1.82^cd^ | 0.04^c^ | 0.09^ef^ | 12.6^d^ | 0.44^cd^ | 0.01^d^ | 15.0^de^ |
|  | Root | 0.07^c^ | 3.69^b^ | 0.07^b^ | 0.41^b^ | 9.83^d^ | 0.97^b^ | 0.04^b^ | 15.1^de^ |
|  | Stem | 0.08^c^ | 4.77^a^ | 0.08^b^ | 0.34^c^ | 20.2^c^ | 0.27^d^ | bDL | 25.8^c^ |
|  | Leaf | bDL | 4.82^a^ | 0.04^c^ | 0.22^de^ | 27.5^b^ | 1.23^a^ | 0.04^b^ | 33.8^b^ |

Mean values (n=3) ± SD; identical letters (a, b, c..) followed by values denote no significant (p = 0.05) difference in columns according to Tukey's HSD test (ANOVA)

bDL – below detection limit
